# Wnt/β-catenin pathway regulates MGMT gene expression in cancer and inhibition of Wnt signalling prevents chemoresistance

**DOI:** 10.1038/ncomms9904

**Published:** 2015-11-25

**Authors:** Malin Wickström, Cecilia Dyberg, Jelena Milosevic, Christer Einvik, Raul Calero, Baldur Sveinbjörnsson, Emma Sandén, Anna Darabi, Peter Siesjö, Marcel Kool, Per Kogner, Ninib Baryawno, John Inge Johnsen

**Affiliations:** 1 Department of Women's and Children's Health, Childhood Cancer Research Unit, Karolinska Institutet, Stockholm S-17176, Sweden; 2 Department of Pediatrics, University Hospital of North Norway, Tromsø N-9038, Norway; 3 Department of Medical Biology, University of Tromsø, Tromsø N-9037, Norway; 4 Department of Clinical Sciences Lund, Glioma Immunotherapy Group, Division of Neurosurgery, Lund University, Lund S-22185, Sweden; 5 Division of Pediatric Neuro-Oncology, German Cancer Research Center, DKFZ, Heidelberg 69120, Germany; 6 Department of Stem Cell and Regenerative Biology, Harvard University, Cambridge, Massachusetts 02138, USA; 7 Center for Regenerative Medicine and the Cancer Center, Massachusetts General Hospital, Boston, Massachusetts 02114, USA; 8 Harvard Stem Cell Institute, Cambridge, Massachusetts 02138, USA

## Abstract

The DNA repair enzyme O6-methylguanine-DNA methyltransferase (MGMT) is commonly overexpressed in cancers and is implicated in the development of chemoresistance. The use of drugs inhibiting MGMT has been hindered by their haematologic toxicity and inefficiency. As a different strategy to inhibit MGMT we investigated cellular regulators of MGMT expression in multiple cancers. Here we show a significant correlation between Wnt signalling and MGMT expression in cancers with different origin and confirm the findings by bioinformatic analysis and immunofluorescence. We demonstrate Wnt-dependent MGMT gene expression and cellular co-localization between active β-catenin and MGMT. Pharmacological or genetic inhibition of Wnt activity downregulates MGMT expression and restores chemosensitivity of DNA-alkylating drugs in mouse models. These findings have potential therapeutic implications for chemoresistant cancers, especially of brain tumours where the use of temozolomide is frequently used in treatment.

## Abstract (web summary)

The high expression of the DNA repair enzyme O6-methylguanine DNA methyltransferase (MGMT) often confers resistance to chemotherapy in several cancers. In this study, the authors propose the inhibition of the Wnt signalling pathway as an alternative strategy to modulate MGMT expression and sensitize tumours to chemotherapy.

## Graphical abstract

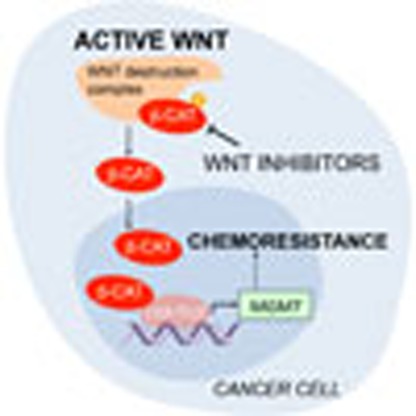

One of the major hurdles in cancer treatment is development of resistance against chemotherapeutic drugs. MGMT efficiently removes alkylating lesions at the O^6^ position of guanine and treatment failure caused by the ability of MGMT to repair DNA damage induced by DNA alkylators or chloroethylating agents is frequently observed^1^. Specifically, temozolomide, widely used in treatment of malignant brain tumours, has low effectiveness in tumours with elevated MGMT activity^2^,^3^. Unfortunately, systemic clinical use of MGMT inhibitors has been restricted mainly because of an increase in haematologic toxicity to DNA alkylators^4^,^5^, and failure in restoring temozolomide sensitivity to temozolomide-resistant glioblastoma multiforme^6^.

MGMT is an evolutionary conserved and ubiquitously expressed enzyme that is regulated by multiple mechanisms including epigenetic silencing of the MGMT gene by promoter methylation, frequently observed in gliomas and colon cancer^7^. Also histone modifications and aberrant expression of transcriptional activators and repressors, as well as microRNAs binding to the 3′-untranslated region of the MGMT gene contribute to the differential expression levels of MGMT in various tumours and normal tissues^7^. Given that expression of MGMT is regulated by multiple molecular mechanisms we searched for cellular regulators of MGMT that can be specifically targeted to lower the levels of MGMT in tumour cells and re-sensitize these tumours to chemotherapeutic drugs. We show that activation of the canonical Wnt/β-catenin signalling cascade induce MGMT expression, and that inhibition of Wnt signalling augment the effects of alkylating drugs and restore chemosensitivity in different cancers.

## Results

### Wnt/β-catenin activation correlates with MGMT expression

**Figure 1:**
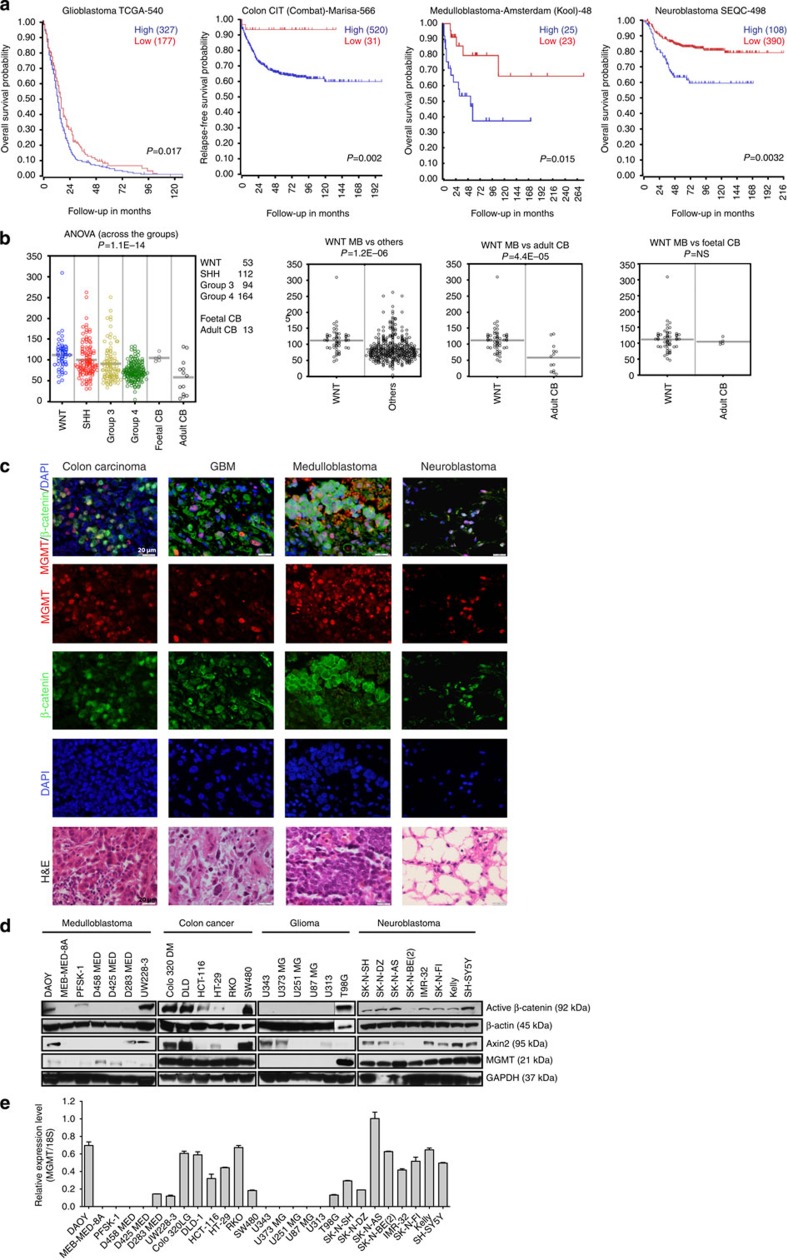
Activation of canonical Wnt/β-catenin correlates with MGMT expression in tumours of different origins. (**a**) Kaplan–Meier survival estimates of high/low MGMT expression in colon carcinoma, glioblastoma, medulloblastoma and neuroblastoma. The Kaplan scanning tool in the R2 genomics analysis and visualization platform (r2.amc.nl) was used to check for MGMT mRNA expression in the different cancer types. All MGMT expression data were scanned to find the most optimal cut-off between high and low MGMT gene expression and the log-rank test that gave the lowest *P*-value were calculated to search for significant differences between tumour samples expressing high and low MGMT mRNA levels. *P*-values were corrected for multiple testing (one-way ANOVA). (**b**) MGMT expression in medulloblastoma molecular subgroups (Wnt, Shh, Group 3 and Group 4 (ref. 36). MGMT expression is significantly (*P*=1.2 × 10^−6^) higher in the Wnt molecular subgroup of medulloblastoma compared with other medulloblastoma subgroups and normal cerebellum but not foetal cerebellum. (**c**) Expression of MGMT and active β-catenin are localized to the same cells in clinical tumour samples from medulloblastoma, glioma, neuroblastoma and colon carcinoma. Shown are β-catenin (green), MGMT (red), DAPI (blue), overlays (top) and hematoxylin and eosin (H&E) staining. Scale bar, 20 μm. (**d**,**e**) MGMT mRNA expression correlates with active β-catenin in cell lines derived from different cancers. A correlation between active β-catenin and MGMT mRNA expression was detected in the majority of the 27 cell lines except for the sPNET cell line PFSK-1 which expressed active β-catenin but no MGMT mRNA, and RKO, D283 MED and SK-N-BE(2) cells expressing MGMT but low levels of active β-catenin. No active β-catenin or MGMT were detected in five out of six glioma cell lines tested. The deficiency of MGMT may also be caused by MGMT promoter methylation commonly seen in glioma cell lines. For all cell lines, protein extracts were subjected to western blotting using antibodies to detect activated desphosphorylated form of β-catenin, Axin 2 that is induced by canonical Wnt/β-catenin signalling and MGMT. Quantitative RT–PCR was used to detect MGMT mRNA levels. For the MGMT mRNA means with s.d. of triplicates are displayed.

To search for cellular regulators of MGMT as an alternative approach to inhibit expression of MGMT in tumour cells we used gene ontology analysis to test for aberrantly expressed genes or signal transduction cascades in cancers with elevated expression of MGMT. Analysis of expression cohorts of tumours with neural origin showed that high MGMT expression levels correlate with poor survival in adult gliomas and childhood neuroblastoma, whereas in medulloblastoma high levels of MGMT was significantly correlated to the Wnt molecular subgroup with high frequency of mutations in Wnt signalling key molecules (Fig. 1a,b, Supplementary Fig. 1). Moreover, in colon cancer, where aberrant Wnt signalling is common^8^, high expression of MGMT correlated with poor prognosis (Fig. 1a, Supplementary Fig. 1). Pathway-specific gene-expression profiling to search for regulators of MGMT expression showed gene-expression signatures that associated with Wnt signalling in colon cancer, neuroblastoma, glioma, as well as for the Wnt-driven medulloblastoma subgroup (Supplementary Fig. 2). K-means clustering of Wnt gene-expression profiles identified subgroups expressing significantly higher levels of MGMT (Supplementary Figs 2a–d and 3a–d). Further, immunofluorescence analysis on human tumour tissues showed co-localization of nuclear β-catenin and MGMT in subtypes of colon cancer, glioma, medulloblastoma and neuroblastoma (Fig. 1c). Co-localization of β-catenin and MGMT was also observed in HT-29 adenocarcinoma xenografts and in lower crypt cells of normal colon (Supplementary Fig. 4). We also detected a correlation between β-catenin as shown by western blots against the active form of β-catenin dephosphorylated on Ser37 or Thr41 and the downstream effector Axin 2 and MGMT expression in the majority of cancer cell lines derived from these cancers (Fig. 1d,e, Supplementary Fig. 7a).

### Wnt/β-catenin regulates MGMT expression

**Figure 2:**
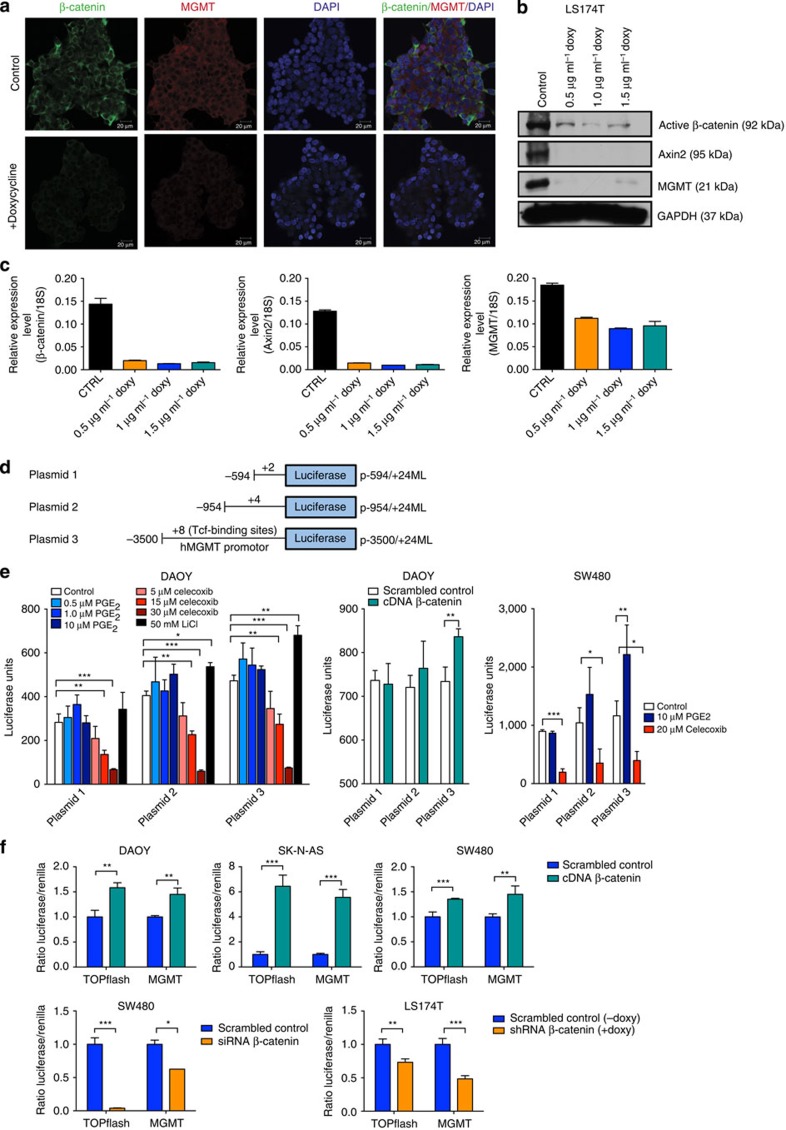
Wnt/β-catenin regulates the expression of MGMT. (**a**,**b**) LS174T cells stably transfected with a Tet-inducable shRNA against β-catenin suppressed MGMT expression upon doxycycline stimulation^9^ (**a**) Immunofluorescence stained LS174T cells treated with 1.5 μg ml^−1^ doxycycline compared with untreated cells. Scale bar, 20 μm. (**b**,**c**) Protein and mRNA expression of β-catenin, Axin 2 and MGMT in cellular extracts from LS174T cells treated with the indicated concentrations of doxycycline (doxy), and protein expression was determined by western blotting. mRNA expression was measured with quantitative RT–PCR, means with s.d. of triplicates are displayed. (**d**–**f**) β-catenin activates MGMT through Tcf/Lef binding located in the hmMGMT 5′-flanking regulatory region. (**d**) Schematic presentation of luciferase reporter plasmids (p-594/+24 ML, p-954/+24 ML and p-3500/+24 ML) with indicated numbers of putative Tcf/Lef-binding sites. Numbers in plasmid names refer to the amount of TCF/LEF-binding sites: +2 (plasmid 1), +4 (plasmid 2) and +8 (plasmid 3). (**e**) DAOY medulloblastoma cells were transiently transfected for 24 h with the MGMT promotor plasmids before treatment with increasing concentrations of either celecoxib, PGE_2_ or LiCl for another 24 h (one-way ANOVA with Bonferroni post test: plasmid 1: *P*<0.0001, plasmid 2: *P*<0.0001, plasmid 3; *P*<0.0001, *P<0.05, **P<0.01, ***P<0.001). DAOY cells were co-transfected with pβ-catSPORT6 expressing β-catenin cDNA and the MGMT promoter plasmids for 48 h before luciferase activity measurements (*t*-test, *P*=0.0092 for plasmid 3). SW480 colon cells were transiently transfected for 24 h with the MGMT promoter plasmids before treatment with celecoxib or PGE_2_ for 24 h (one-way ANOVA with Bonferroni post test: plasmid 1: *P*=0.0007, plasmid 2: *P*=0.0027, plasmid 3: *P*=0.0003, *P<0.05, **P<0.01, ***P<0.001). (**f**) Transfection of DAOY, SK-N-AS, SW480 and LS174T cells with pβ-catSPORT6 or β-catenin siRNA regulates luciferase activity of TOPflash and p-3500/+24 ML (plasmid 3). Luciferase activities are expressed as mean±s.d. of triplicate in one representative experiment (each experiment was repeated two to three times). DAOY, *t*-test: TOPflash *P*=0.0036, MGMT *P*=0.004; SK-N-AS *t*-test: TOPflash *P*=0.0005, MGMT *P*=0.0002; SW480 one-way ANOVA with Bonferroni post test: TOPflash *P*<0.0001, MGMT *P*=0.0003, *P<0.05, **P<0.01, ***P<0.001; LS174T *t*-test TOPflash *P*=0.0014, MGMT *P*<0.0001. For all experiments means with s.d. of triplicates are shown.

To investigate if Wnt signalling is involved in the regulation of MGMT expression, we genetically blocked the activity of Wnt signalling using shRNA that render the Wnt signalling activity within cancer cells. For this purpose we used the LS174T colon carcinoma cell line which is stably transfected with an inducible β-catenin shRNA and downregulates β-catenin expression following addition of doxycycline^9^. Knockdown of β-catenin in LS174T cells inhibited MGMT expression (Fig. 2a–c, Supplementary Fig. 7b).

To further investigate the regulatory influences of β-catenin on MGMT transcription we analysed the 5′-flanking region of the *hmMGMT* gene for putative Tcf/Lef transcription factor-binding sites and detected eight putative binding sites within the MGMT promoter/enhancer (Supplementary Fig. 5). Transfection experiments using the MGMT-5′ regions containing different numbers of Tcf/Lef-binding sites cloned into luciferase reporter plasmids (Fig. 2d) showed an enhancement of luciferase activity with increasing numbers of Tcf/Lef-binding sites upon activation of β-catenin by inhibition of GSK-3β using LiCl^10^ or by overexpression of β-catenin (Fig. 2e). Activation of Wnt signalling with prostaglandin E_2_ (PGE_2_) (ref. 11) induced an increase in luciferase activity, while inhibition of PGE_2_ production with the cyclooxygenase-2 (Cox-2) inhibitor celecoxib showed a concentration-dependent reduction (Fig. 2e). Similarly, overexpression (Fig. 2f) or targeted knockdown (Fig. 2f) of β-catenin lead to an increase or decrease, respectively, of the MGMT promoter in SK-N-AS neuroblastoma, DAOY medulloblastoma, SW480 and LS174T colon carcinoma cells (Fig. 2f). These results further validate that the canonical Wnt signalling cascade directly regulates the expression of MGMT.

### Wnt inhibition augments temozolomide-mediated chemotherapy

**Figure 3:**
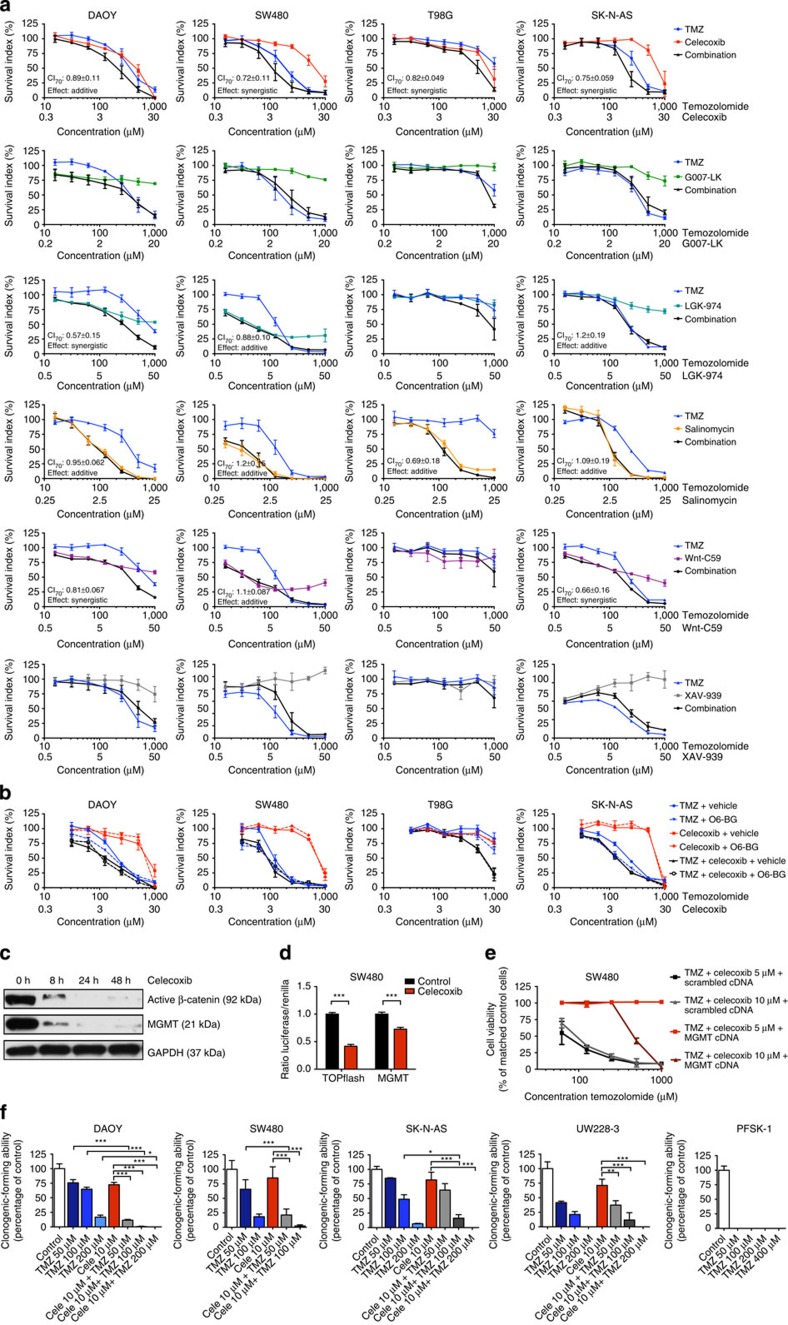
Pharmacological inhibition of Wnt/β-catenin signalling augments the cytotoxic effects of temozolomide. (**a**) Cell viability of cancer cell lines treated with increasing concentrations of different Wnt inhibitors and temozolomide (TMZ). Cell growth was assessed by FMCA or WST-1 after 72 h. All drugs were combined with a fixed molar ratio: TMZ:Celecoxib, 33:1; TMZ:G007-LK, 50:1; TMZ:LGK974, 20:1; TMZ:Wnt-C59, 20:1; TMZ:Salinomycin, 40:1; and TMZ:XAV-939, 20:1. Combination index (CI) at IC_70_ was calculated by the median-effect method. Synergism and antagonism are defined as a CI mean significantly lower/higher than 1 with one-sample *t*-test (*P*<0.05). The combinations with G007-LK and XAV-939 (in all tested cell lines) and with LGK974 and Wnt-C59 in T98G could not be analysed by the median-effect method since the single drug effect did not achieve a full dose–response curve. Each concentration was tested in duplicate and the experiment was repeated at least three times. Values are mean±s.e.m. (**b**) Cell viability of cancer cell lines pre-treated with or without the MGMT inhibitor O6-BG (25 μM), and increasing concentrations of TMZ and celecoxib. Data represent the mean±s.d. of three or four experiments (*t*-test on log IC_50_ with and without O6-BG pre-treatment, DAOY TMZ *P*=0.0237, SW480 TMZ *P*=0.0073, SK-N-AS TMZ *P*=0.0007). (**c**) Western blotting of cellular extracts from SW480 cells treated with 10 μM celecoxib for 0–48 h. (**d**) Treatment with celecoxib 30 μM in SW480 regulates luciferase activity of TOPflash and p-3500/+24 ML (plasmid 3). Luciferase activities are expressed as mean±s.d. of triplicate, experiment was repeated twice. (**e**) Overexpression of MGMT overcomes the cytotoxic effects of TMZ induced by celecoxib. SW480 cells were transfected with MGMT cDNA (pMGMTSPORT6) or scrambled control, left for 24 h and then treated with celecoxib and increasing concentrations of TMZ for 48 h. Data represent the mean±s.e.m. (**f**) Clonogenic capacity of cancer cell lines treated with celecoxib (Cele) and TMZ. Cells were then incubated in drug-free medium for 7–14 days and colonies were counted. Each experimental point was performed in triplicate and repeated three times. Values are mean±s.d. One-way ANOVA, *P<0.05, **P<0.01, ***P<0.001).

Next, we tested a panel of Wnt signalling inhibitors in combination with the DNA-alkylating drug temozolomide on colon carcinoma, medulloblastoma, neuroblastoma and glioma cell survival. The agents tested were the non-specific Wnt signalling inhibitor celecoxib^10^,^12^,^13^, the Porcupine inhibitors Wnt-C59 and LGK974, the tankyrase/Axin 1 inhibitors XAV-939 and G007-LK (refs 14, 15) and salinomycin, which inhibits signalling by blocking phosphorylation of the Wnt co-receptor lipoprotein receptor-related protein 6 (ref. 16). Except for G007-LK and XAV-939, all Wnt inhibitors augmented the cytotoxic effects of temozolomide in the majority of the tested cancer cell lines (Fig. 3a). Celecoxib was the most profound compound inducing either an additive or synergistic effect on cell cytotoxicity in combination with temozolomide in all investigated cell lines. Celecoxib was therefore selected for further analysis.

**Figure 4:**
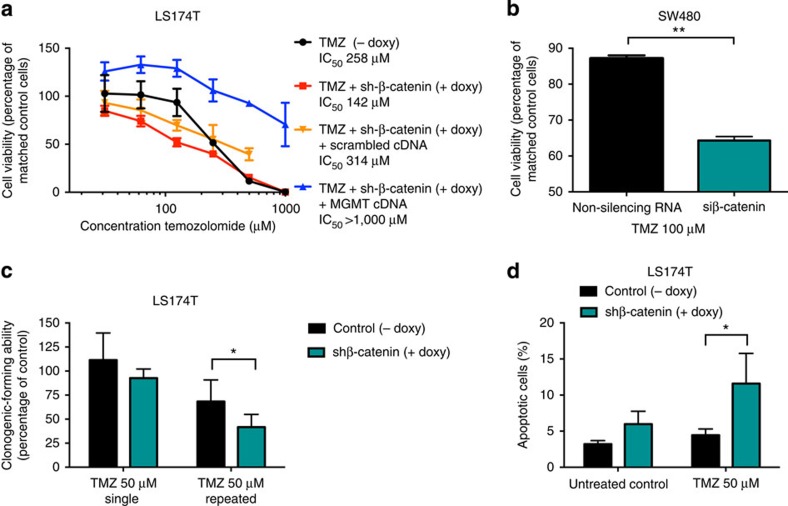
Genetical inhibition of Wnt/β-catenin signalling augments the cytotoxic effects of temozolomide. (**a**,**b**) Inhibition of β-catenin expression by shRNA or siRNA sensitizes LS174T (**a**) and SW480 (**b**) colon carcinoma cells to temozolomide. LS174T cells were grown in medium containing ±1 mg ml^−1^ doxycycline (doxy) and treated with increasing concentrations of temozolomide (TMZ) twice (at 0 and 48 h) for totally 96 h. Doxy-induced shRNA knockdown of β-catenin expression significantly augment the cytotoxic effect of TMZ (*t*-test on log IC_50_, *P*=0.0003). Overexpression of MGMT reversed the cytotoxic effect of TMZ caused by β-catenin knockdown (*t*-test on log IC_50_, *P*=0.0491). Each concentration was tested in triplicate and the experiments were repeated twice. Values are mean±s.e.m. and presented as the percentage of matched control cells. Only depletion of β-catenin inhibited cell growth to 46±5.8% (mean±s.e.m.) of untreated control. (**b**) SW480 cells were transiently transfected with siRNA against β-catenin and subsequently treated with 100 μM TMZ for 48 h. Significant growth inhibition was observed in cells treated with a combination of siRNA against β-catenin and TMZ compared with only TMZ (*t*-test, *P*=0.002). The experiment was repeated twice. Values are mean±s.e.m. (**c**) Clonogenic capacity of LS174T cells ±1 μg ml^−1^ doxy to induce shRNA against β-catenin and treated with 50 μM TMZ, as a single treatment or repeated treatment. The TMZ treatment was significantly more efficient in the shRNA-induced LS174T cells (*t*-test, *P*=0.032). Each experimental point was performed in triplicate. The experiment was repeated with similar results. Mean±s.d. are displayed. (**d**) β-catenin knockdown significantly increases TMZ-induced apoptosis in LS174T cells. LS174T cells were incubated with ±1 μg ml^−1^ doxy to induce shRNA against β-catenin and treated with 50 μM TMZ for 48 h (*t*-test, *P*=0.036). Apoptosis was analysed by flow cytometry measurement of cells in sub-G0 phase. The experiment was repeated three times. Values are mean±s.d.

We next compared the effect of celecoxib to restore temozolomide sensitivity in cells expressing high levels of MGMT with the MGMT-specific inhibitor O6-Benzylguanine (O6-BG). O6-BG and celecoxib induced similar cytotoxic effects on the cells in combination with temozolomide and no additional cytotoxic effects of temozolomide were observed when O6-BG was used in combination with celecoxib and temozolomide (Fig. 3b). Moreover, celecoxib downregulated both the expression of endogenous β-catenin and MGMT (Fig. 3c, Supplementary Fig. 7c), as well as transcriptional activation induced by β-catenin (Fig. 3d), whereas forced MGMT overexpression by transfection of MGMT cDNA abrogated the cytotoxic effects seen with celecoxib and temozolomide (Fig. 3e). To further test whether celecoxib can restore temozolomide sensitivity to temozolomide-resistant cancer cells *in vitro* we first treated cells expressing either low or high levels of MGMT with increasing concentrations of temozolomide and measured the tumourigenic capacity using clonogenic assays. MGMT expression levels corresponded with treatment efficiency (IC_50_>100 μM for DAOY, UW228-3, SK-N-AS and SW480; IC_50_<50 μM for the MGMT-negative cell lines PFSK-1, Figs 3f and 1d). However, when celecoxib was used in combination with temozolomide we observed a significant restoration of temozolomide chemosensitivity in all MGMT-positive cell lines (Fig. 3f). Similar, but less efficient inhibitory effects were observed using the Porcupine inhibitors Wnt-C59 and LGK974 or the tankyrase/Axin 1 inhibitor G007-LK (Supplementary Fig. 6). To investigate the mechanism of the demonstrated cytotoxic effect we used the LS174T colorectal carcinoma cell line with Tet-inducible shRNA against β-catenin^9^. A significant increased suppression of cell growth was observed in LS174T cells treated with temozolomide where β-catenin was depleted compared with control cells. This synergistic effect could be reversed when adding MGMT cDNA to the β-catenin-depleted cells (Fig. 4a). Similarly, siRNA inhibition of β-catenin significantly restored temozolomide cytotoxicity in SW480 cells (Fig. 4b). Moreover, a significant inhibition of clonogenic capacity was observed in β-catenin knockdown cells treated with temozolomide compared with temozolomide-treated wild-type cells (Fig. 4c). This was accompanied by an increase of apoptosis in β-catenin knockdown cells treated with temozolomide compared with LS174T treated with temozolomide only (Fig. 4d).

### Inhibition of Wnt restores temozolomide sensitivity *in vivo*

**Figure 5:**
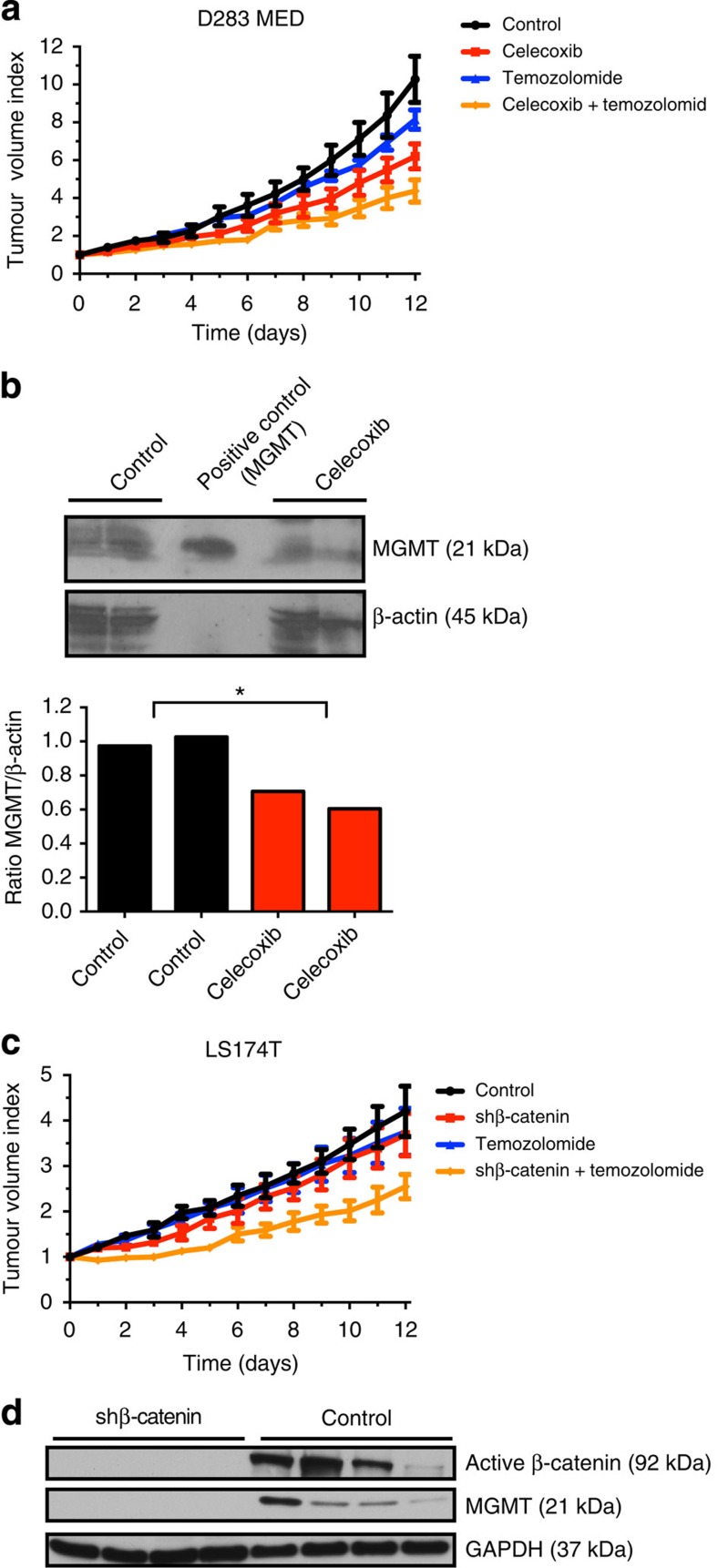
Inhibition of Wnt/β-catenin in combination with temozolomide reduces tumour growth *in vivo*. (**a**) A combination of temozolomide and celecoxib significantly impairs the growth of established human medulloblastoma xenografts in NMRI nu/nu mice. Mice were engrafted with 7 × 10^6^ D283 MED cells subcutaneously and randomized to receive either celecoxib (90 mg kg^−1^; *n*=12) through daily oral gastric feeding, temozolomide (7.5 mg kg^−1^; *n*=9; days 1–5), a combination of celecoxib and temozolomide (*n*=9) or no treatment (*n*=10), starting at the appearance of palpable tumours of approximately 0.10 ml (mean 0.13 ml). Celecoxib augments the inhibitory effect of temozolomide on medulloblastoma growth *in vivo*, as shown by the TVI (at day 12, celecoxib: TVI=6.2, *P*<0.0001; temozolomide: TVI=8.1, not significant; combination: TVI=4.4 versus 10.3 in untreated controls, *P*<0.0001, TVI, two-way ANOVA). (**b**) Western blot of protein extracts isolated from celecoxib- and vehicle-treated xenograft tumours. Celecoxib downregulated the expression of MGMT *in vivo*. Protein expression was assessed with densitometry (*t*-test, *P*=0.0266). (**c**) A combination of β-catenin knockdown and temozolomide inhibits the growth of established colon adenocarcinoma xenografts *in vivo*. NMRI nu/nu mice were engrafted with 10 × 10^6^ LS174T cells subcutaneously and randomized (10 mice each group) to receive 2 mg ml^−1^ doxycycline to induce shRNA against β-catenin, 12.5 mg kg^−1^ temozolomide i.p. from day 1 to day 5 and day 8 to day 12, a combination of temozolomide and doxycycline or vehicle only. No differences were observed in LS174T xenograft tumour growth treated with doxycycline or temozolomide as single treatment, whereas a combination of β-catenin knockdown and temozolomide induced significant inhibition of tumour growth at day 7 of treatment until the end of treatment (at day 12: sh-β-catenin: TVI=3.7, not significant; temozolomide: TVI=3.8, not significant; combination: TVI=2.5 versus 4.2 in untreated controls, *P*<0.0001; combination versus shβ-catenin, *P*<0.01; combination versus temozolomide, *P*<0.01, two-way ANOVA). LS174T shRNA sequence directed against β-catenin: 5′-GATCCCGTGGGTGGTATAGAGGCTCTTCAAGAGAGAGCCTCTATACCACCCACT TTTTGGAAA-3′ (**d**) Western blot of protein extracts isolated from LS174T xenograft tumours confirm downregulation of active β-catenin and MGMT.

Celecoxib was selected for further analysis based on its ability to inhibit Wnt signalling^17^, downregulate MGMT expression (Figs 2e and 3c,d) and induce synergistic toxicity on tumour cells both in combination with temozolomide and several other cytotoxic drugs used as a first-line treatment of different cancers (Fig. 3a and Supplementary Table 1). Celecoxib and other non-steroidal anti-inflammatory drugs, approved by the FDA and EMEA, have proven anti-tumourigenic effects in preclinical models and reduce the incidence and severity of various human cancers^10^,^12^,^13^,^17^,^18^,^19^,^20^. We therefore next used pharmacological and genetic inhibition to investigate the effect of β-catenin blockade on tumour cell sensitivity to temozolomide *in vivo*. For this purpose we used two cell lines expressing either low (D283 MED)^21^ or high levels of MGMT (LS174T; Fig. 1d). Administration of temozolomide or celecoxib alone to nude mice carrying established D283 MED medulloblastoma xenografts resulted in significant tumour growth inhibition after 12 days of treatment and the tumour volume index (TVI) was reduced by 40 and 20%, respectively, when compared with untreated controls, at the end of treatment (Fig. 5a). In combination, celecoxib and temozolomide reduced tumour growth by 57% (Fig. 5a). The expression of MGMT was repressed in celecoxib-treated xenografts as compared with control xenograft tumours (Fig. 5b, Supplementary Fig. 7d). We also tested the effect of β-catenin knockdown using LS174T cells with Tet-inducible shRNA against β-catenin. No differences in tumour growth were observed in mice with wild-type β-catenin levels, in mice receiving doxycycline to induce β-catenin depletion, or mice treated with temozolomide only (TVI reduction with 16 and 9%, respectively; Fig. 5c). However, a significant inhibition of tumour growth was observed in mice with β-catenin-depleted tumours treated with temozolomide (TVI was reduced with 60%; Fig. 5c). Expression of MGMT was completely blocked in β-catenin-depleted xenografts as compared with control xenograft tumours (Fig. 5d, Supplementary Fig. 7e). Together these results suggest that targeting β-catenin restores temozolomide chemosensitivity in cancer cells expressing MGMT.

## Discussion

In this study we provide an alternative strategy for targeting chemoresistant cancers by modulating MGMT protein expression through canonical Wnt cascade inhibition. Given the importance of Wnt signalling during embryonic development and the high fidelity of accurate DNA replication in stem cells^22^ it is equitable that Wnt signalling may regulate the activity of DNA repair enzymes. MGMT is a suicide enzyme that removes O^6^-guanosine alkylation adducts caused by alkylation agents in a one-step reaction that restores the guanosine residue to its unchanged state but renders MGMT inactive^23^. Hence, the expression level of MGMT is therefore fundamental for accurate DNA repair.

Although it remains to be evaluated, the indirect inhibition of MGMT via suppression of Wnt activity will most likely avoid some of the haematological toxicities observed by systemic administration of small-molecule inhibitors of MGMT. The temozolomide dosages (7.5 and 12.5 mg kg^−1^) used in the xenograft studies are equivalent to a human dose of 90 and 150 mg m^−2^, respectively^24^. For the treatment of brain tumours in adults an initiation regimen of 150 mg m^−2^ temozolomide followed by a maintenance doses of 200 mg m^−2^ once daily is recommended. However, in humans approximately 20% of temozolomide detected in the blood plasma (area under the concentration-time curve (AUC) 30.1 s.e.m. 6.1 mg/l-h in humans receiving 200 mg m^−2^ as compared with AUC 6.1 s.e.m. 1.2 mg/l-h in cerebrospinal fluid, derived from 35 patients with a total of 227 plasma and 47 CSF samples was analysed) is present in the cerebrospinal fluid^25^. Although we did not perform pharmacokinetics for temozolomide in our experiments the systemic AUC as interpolated from others^6^,^26^ suggest that the systemic AUC for temozolomide in mice receiving 12.5 or 7.5 mg kg^−1^ to be approximately 7.6 and 4.5 mg/l-h, respectively. This indicates that the selected temozolomide doses used for the xenografts experiments correspond to the obtainable AUC observed in the cerebrospinal fluids. In conclusion, our data supports the use of inhibitors of Wnt signalling and temozolomide in combination as a treatment option for cancer patients expressing high levels of MGMT in their tumour.

## Methods

### Chemicals

Celecoxib (Pfizer, Täby, Sweden), dimethyl-PGE_2_ (dmPGE_2_), cyclophosphamide (given as the active metabolite 4-hydroxycyclophosphamide), temozolomide, doxycycline, XAV-939, salinomycin (all from Sigma-Aldrich, Solna, Sweden), O6-BG, Wnt-C59 and LGK974 (Cayman Chemical, Ann Arbor, MI, USA) were dissolved in dimethyl sulfoxide (Sigma-Aldrich). Cisplatin, irinotecan, doxorubicin and vincristine were purchased from the local pharmacy (Apoteket AB, Sweden) and diluted according to manufactures instructions. G007-LK was a kind gift from Dr Krauss, University of Oslo, Norway^14^,^27^. The mTOR inhibitors rapamycin (Sirolimus, LC Laboratories, Woodburn, MA, USA) and CCI-779 (Temsirolimus, a kind gift from Wyeth Pew River, NY, USA) were dissolved in 99.5% ethanol. LiCl was dissolved in H_2_O. All inhibitors/activators were further diluted in OptiMEM (Gibco BRL, Sundbyberg, Sweden) to the desired *in vitro* concentration. Temozolomide for the *in vivo* studies was supplied by the local pharmacy (Apoteket AB). For *in vivo* use of celecoxib and temozolomide in the D283 xenograft study, the stock was prepared as a suspension in a vehicle fluid consisting of 0.5% methylcellulose (w/v; Sigma-Aldrich) and 0.1% Tween 80 (v/v; Sigma-Aldrich) in sterile water. For the LS174T xenograft study, temozolomide was dissolved in NaCl and doxycycline in water.

### Cell lines

Cell lines used were kindly provided by Dr T. Pietsch (University of Bonn Medical Center, Bonn, Germany), Dr C. Redfern (Northern Institute for Cancer Research, Newcastle University, Newcastle, UK), Dr M. Nister, Dr Söderberg-Naucler and Dr M. Farnebo (Karolinska Institutet, Stockholm, Sweden) and Dr H. Clevers (Hubrecht Institute and University Medical Center Utrecht, Utrecht, Holland), except from DAOY (D324 MED), D283 MED, HT-29, PFSK-1, T98G and SK-N-AS that were purchased from ATCC. The cell lines were cultured in Dulbecco's Modified Eagle's Medium (DMEM; MEB-MED-8A, U373 MG, U251 MG ACII, U87 MG, U343, U313, HCT-116, RKO, SK-N-AS), minimum essential media (MEM; DAOY, D283 MED, T98G), RPMI-1640 (PFSK-1, Colo-320-DM, DLD-1, HT-29, SW480, LS174T), Richter's improved MEM with zinc/DMEM (IMEMZO/DMEM; (D425MED and D458 MED), DMEM/F12 (UW228-3)). Medium was supplemented with 10% heat-inactivated foetal bovine serum, 2 mM L-glutamine, 100 IU ml^−1^ penicillin G, and 100 μg ml^−1^ streptomycin (Life Technologies, Stockholm, Sweden) at 37 °C in a humidified 5% CO_2_ atmosphere. All media were purchased from Gibco BRL. LS174T was grown in doxycycline in indicated concentrations for at least 3 days before experimental use with silenced β-catenin. All *in vitro* studies were carried out in OptiMEM supplemented with antibiotics and L-glutamine.

### Gene-expression profiling and TCF/LEF-binding sites

Gene-expression profiling on glioblastoma, colon carcinoma, medulloblastoma and neuroblastoma were performed as described previously^28^,^29^. Data analyses (K-means clustering, Kaplan–Meier survival curves and MGMT expression analyses) on publicly available gene-expression data sets were performed using the R2 microarray analysis and visualization platform (http://r2.amc.nl). TCF/LEF-binding sites within the 5′-flanking promoter region of the MGMT gene were searched for with BLAST (Basic Local Alignment Search Toolvate) programs (http://www.ncbi.nlm.nih.gov) and PROMO (http://alggen.lsi.upc.es;) (ref. 30).

### Immunohistochemistry

Formalin-fixed and paraffin-embedded tissue sections were deparaffinized in xylene, rehydrated in graded alcohols and washed in phosphate-buffered saline (PBS). After antigen retrieval in sodium citrate buffer (pH 6) in a microwave oven sections were blocked with 5% goat serum (Jackson Immunoresearch, Fisher Scientific, Gothenburg, Sweden) and incubated for 48 h at 4 °C with the primary antibody (mouse monoclonal MGMT, prediluted, 54306 Abcam, Cambridge Science Park, Cambridge, UK). Thereafter, sections were incubated for 30 min at room temperature with anti-mouse Alexa Fluor 568 or 594 conjugate. Similarly, for β-catenin detection, sections were incubated for 48 h at 4 °C with polyclonal rabbit anti-β-catenin (Cell Signaling Technology #8480) or rabbit anti-β-catenin (4 μg ml^−1^, Abcam), followed by detection with goat-anti-rabbit Alexa Fluor 488 conjugate. Sections were mounted with Dapi-fluoromount-G (Southern Biotech) mounting medium. Parallell tissue sections were stained with hematoxylin and eosin.

For immunofluorescence studies, LS174T cells were treated with 1 μg ml^−1^ in doxycycline for 7 days and then grown on chamber slides (Nunc, Roskilde, Denmark) for 72 h. Thereafter the cells were washed with PBS and fixed with 2% paraformaldehyde for 30 min and cold methanol (70%) for 15 min. After washing, cell cultures were incubated with anti-MGMT antibody and anti-β-catenin antibody as described above. Pictures were taken on Zeiss LSM 780 microscope using the same acquisition settings for all cell cultures.

### Cytotoxicity and clonogenic assay

The effects of Wnt inhibitors in combination with temozolomide and other conventional chemotherapeutic drugs on cell growth were determined using cell-viability assays fluorometric microculture cytotoxicity assay as previously described^20^,^31^ or WST-1 (Roche Diagnostic, Basel, Switzerland) according to the manufactures description. All concentrations were tested in duplicate or triplicate and the experiments were repeated at least three times. The studies were designed with a fixed molar ratio between the drugs (Temozolomide:Celecoxib, 33:1; Temozolomide:G007-LK, 50:1; Temozolomide:LGK974, 20:1; Temozolomide:Wnt-C59, 20:1; Temozolomide:Salinomycin, 40:1; and Temozolomide:XAV-939, 20:1), intended to be equipotent.

To determine colony formation, DAOY, UW228-3, SK-N-AS, PFSK-1 and SW480 cells were seeded in Cell^+^ six-well plates (Sarstedt, Solna, Sweden) at a concentration of 150–200 cells per dish, in triplicate. Cells were left to attach to the surface for 5 h before treatment with 10 μM celecoxib, 10–20 μM G007-LK, 5–10 μM LGK974 or 10 μM Wnt-C59, 50–400 μM temozolomide, or a combination of temozolomide and one of the Wnt-inhibiting drugs for 48 h, respectively. After 7–14 days of incubation in drug-free medium, cell cultures were rinsed with PBS, fixed in formaldehyde, and stained with Giemsa (Gibco, BRL). Colonies (>75 cells) with 50% plate efficiency were counted manually using a colony counter.

### Immunoblotting

Total cell protein lysates was extracted from cells in RIPA buffer (25 mM Tris (pH 7.8), 2 mM EDTA, 20% glycerol, 0.1% Nonidet P-40 (NP-40), 1 mM dithiothreitol). All protein extraction buffers were supplemented with MiniComplete protease inhibitor cocktail (Roche Diagnostic) and phosphatase inhibitor cocktail 1 (Sigma-Aldrich). The protein concentration was measured using Bradford reagent (Bio-Rad, Sundbyberg, Sweden). Equal quantities were separated by SDS-PAGE, transferred to nylon membranes (Millipore, Sundbyberg, Sweden), and probed with antibodies against active β-catenin (1:1,000, clone 8E7, Millipore, Solna Sweden), Axin2 (1:1,000, Cell Signaling Technology, Beverly, MA, USA), MGMT (1:1,000, Cell Signaling Technology), GADPH (1:10,000, Millipore) and β-actin (1:5,000, Cell Signaling Technology). Anti-rabbit IgG or anti-mouse IgG, conjugated with horseradish peroxidase (Pharmacia Biosciences, Uppsala, Sweden), were used as secondary antibodies. Pierce Super Signal (Pierce, Rockford, IL, USA) was used for chemiluminescent detection. MGMT recombinant protein served as positive control (Assay Designs, Ann Arbor, MI, USA). Quantification of blots were done with densitometry measurements in ImageJ^32^.

### Flow cytometry

To measure sub-G0 cell population, cells were trypsinized, washed with ice-cold PBS, and fixed in 70% ethanol at −20°C. Cells were washed with PBS and incubated with RNase 20 μg ml^−1^ and propidium iodide (PI) 0.5 μg ml^−1^ (Sigma-Aldrich). All analyses were performed with LSR II Flow Cytometer and FACSDiva v.6.1.3 software (BD Biosciences).

### Quantitative real-time RT–PCR analyses

The mRNA expression levels of MGMT, CTNNB1, AXIN2 and an endogenous housekeeping gene encoding for 18S ribosomal RNA as a reference were quantified using TaqMan technology on an ABI PRISM 7500 sequence detection systems (PE Applied Biosystems). Sequence-specific primers and probes were selected from the Assay-on-Demand products (Applied Biosystems), including MGMT (assay ID: Hs01037698_m1), CTNNB1 (Hs00355049_m1), AXIN2 (Hs00610344_m1) and 18S ribosomal RNA (Hs99999901_s1). All gene-expression assays had an FAM reporter dye at the 5′ end of the TaqMan MGB probe, and a non-fluorescent quencher at the 3′ end of the probe.

cDNA of each sample was synthesized from 100 ng of RNA using High capacity RNA-to-cDNA kit (Applied Biosystems). The quantitative real-time RT–PCR was performed in a total reaction volume of 25 μl containing 1 × TaqMan Universal PCR Master Mix, 1 × TaqMan Gene Expression Assays (Applied Biosystems) and 10 μl of cDNA from each sample as a template, in MicroAmp optical 96-well plates covered with MicroAmp optical caps (Applied Biosystems). Samples were heated for 2 min at 50 °C and amplified for 40 cycles of 15 s at 95 °C and 1 min at 60 °C. To establish a standard curve for relative quantification we used cDNA synthesized from 1 μg RNA of the cell lines combined. For each sample, the amount of target mRNA was normalized to the standard curve and then normalized to 18S ribosomal RNA expression. All quantitative real-time RT–PCR experiments included a no template control and were performed in triplicate.

### Transfections and luciferase assays

All transfections were carried out using Lipofectamine 2000 (Invitrogen) according to the manufacturer's instructions. MGMT promoter-activity measurements were achieved using luciferase reporter plasmids that were kindly provided by Dr Bhakat K. Kishor, University of Texas. Construction of plasmids is described elsewhere^33^. Three different lengths of the MGMT promoter coupled to reporter gene were used; the three MGMT promoter constructs contain +1, +3 or +8 putative TCF/LEF-binding sites, respectively (MatInspector). TOPflash luciferase reporter plasmid, M50 Super 8 × TOPFlash was a gift from Randall Moon (Addgene, plasmid #12456 (ref. 34), was used to measure β-catenin-mediated transcriptional activation. Renilla Luciferase Assay System (Promega Biotech AB) was used as control. Briefly, 50,000–250,000 cells (depending on cell type) were seeded on 24-well plates 24 h prior transfection and luciferase expression plasmids (500 ng per well) were transfected into cells. When applicable, after 24 h cells were treated with increasing concentrations of celecoxib, PGE_2_ or LiCl. Cells were collected 48 h after transfection. Luciferase activity was measured using kits from Promega Biotech AB according to the manufacturer-recommended protocols. β-catenin knockdown was achieved using the SignalSilence β-catenin kit (Cell Signaling Technology) according to the manufacturer's instructions. Non-silencing siRNA was used as control (Cell Signaling Technology). Overexpression of β-catenin and MGMT was done with pβ-catSPORT6 expressing β-catenin (Cell Signaling Technology) and pMGMTSPORT6 expressing MGMT (Dharmacon, VWR International, Stockholm, Sweden).

### Nude mice tumour xenografts

Four-to-six-week-old female NMRI nu/nu mice (Scanbur, Stockholm, Sweden) were maintained at five each per cage and were given sterile water and food *ad libitium*. In the first experiment each NMRI nu/nu mouse was subcutaneously injected with 7 × 10^6^ human D283 MED medulloblastoma cells. Mice were randomly assigned into four treatment groups and the drugs were administered as follows: (i) 90 mg kg^−1^ celecoxib (*n*=12) daily by oral gavage, (ii) 7.5 mg kg^−1^ temozolomide (*n*=9) orally via a gastric feeding tube at days 1–5, (iii) 90 mg kg^−1^ celecoxib and 7.5 mg kg^−1^ temozolomide (*n*=9), (iv) no treatment (*n*=10). In the second experiment each mouse was subcutaneously injected with 10 × 10^6^ human LS174T colon carcinoma cells. Mice were randomly assigned into four groups and the mice were treated as follows: (i) 12.5 mg kg^−1^ temozolomide intraperitoneally at days 1–5 and days 8–13 (*n*=9), (ii) 2 mg ml^−1^ doxycycline added to drinking water to knockdown β-catenin expression by shRNA (*n*=8), (iii) a combination of 12.5 mg kg^−1^ temozolomide and 2 mg ml^−1^ doxycycline (*n*=9) and (iv) no treatment (*n*=8). In both experiments each mouse was treated for 12 days and treatment was started on the appearance of palpable tumours. The mean tumour volume at the start of treatment was 0.13 ml for mice injected with D283 MED cells and 0.22 ml for mice carrying LS174T xenografts. Tumours were measured every day and tumour volume was calculated as (width)^2^ × length × 0.44. TVI was calculated using the measured volume divided by the volume measured at start of treatment. At autopsy, tumours were frozen in liquid nitrogen for subsequent analysis. HT-29 colon cancer xenografts were established by inoculation of 10^6^ HT-29 cells intradermally in the abdomen of nude Balb/c mice (Harlan, The Netherlands). At autopsy, the mice were perfused with cold PBS followed by 4% paraformaldehyde and the tumour tissue was excised and further processed for histological analysis. All animal experiments were approved by the regional ethics committee for animal research (approval N304/08 and N391/11) in accordance with the Animal Protection Law (SFS1988:534), the Animal Protection Regulation (SFS 1988:539) and the Regulation for the Swedish National Board for Laboratory Animals (SFS1988:541).

### Statistical analysis

All statistical analyses were performed with GraphPad Prism software (GraphPad Software, San Diego, CA, USA). Calculation of IC_50_ values was done from log-concentration-effect curves in GraphPad Prism. Testing for synergistic or additive effects of combination therapy was performed as previously described according Chou–Talalay method with Combosyn software^20^,^35^. Synergism and antagonism are defined as a mean of the combination index at 70% growth inhibition significantly lower/higher than 1 with one-sample *t*-test (*P*<0.05). For *in vitro* studies, the *t*-test was used to determine whether the mean of a single sample differed significantly from control. To compare several treatment groups, one-way ANOVA with Bonferroni multiple-comparisons tests were used. *P*<0.05 was considered significant. Tumour growth was analysed by two-way ANOVA and the Bonferroni *post hoc* test was used for multiple comparisons between groups.

## Additional information

**How to cite this article:** Wickström, M. *et al.* Wnt/β-catenin pathway regulates MGMT gene expression in cancer and inhibition of Wnt signalling prevents chemoresistance. *Nat. Commun.* 6:8904 doi: 10.1038/ncomms9904 (2015).

## Supplementary Material

Supplementary Information Supplementary Figures 1-7 and Supplementary Table 1
