# Supplementary material for: Wnt/β-catenin pathway regulates MGMT gene expression in cancer and inhibition of Wnt signalling prevents chemoresistance
Source: Nat Commun. 2015 Nov 25;6:8904. doi: 10.1038/ncomms9904 (PMC4674781; doi:10.1038/ncomms9904)
Supplement: Supplementary Information — Supplementary Figures 1-7 and Supplementary Table 1 [file ncomms9904-s1.pdf]

## Supplementary Figures

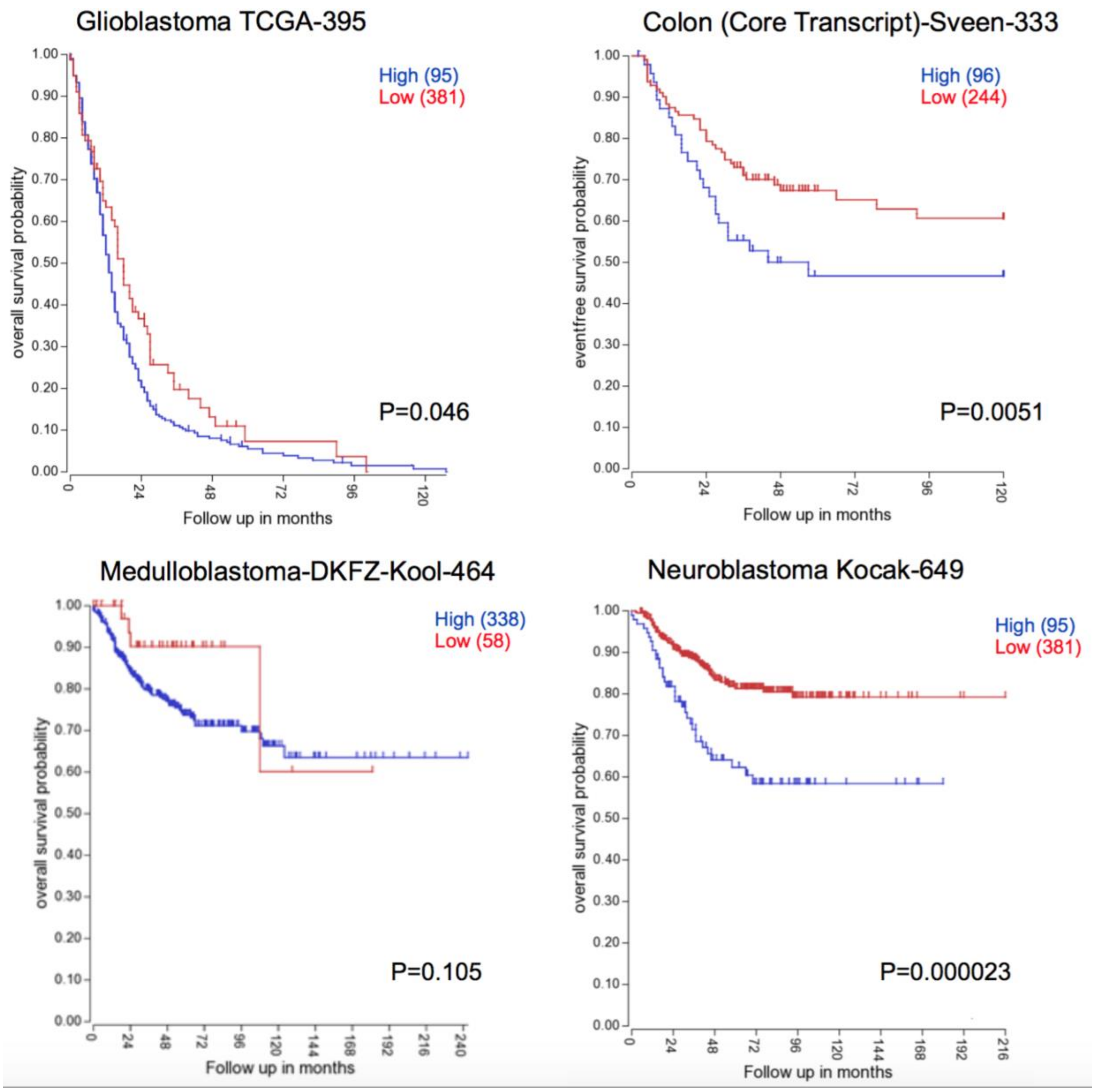


**Supplementary figure 1.** Kaplan-Meier survival estimates of high/low MGMT expression in colon carcinoma, glioblastoma, medulloblastoma and neuroblastoma. The Kaplan scanning tool in the R2 genomics analysis and visualization platform (r2.amc.nl) was used to check for MGMT mRNA expression in the different cancer types. All MGMT expression data were scanned to find the most optimal cut-off between high and low MGMT gene expression and the log rank test that gave the lowest p-value were calculated to search for significant differences between tumour

samples expressing high and low MGMT mRNA levels. P-values were corrected for multiple testing.

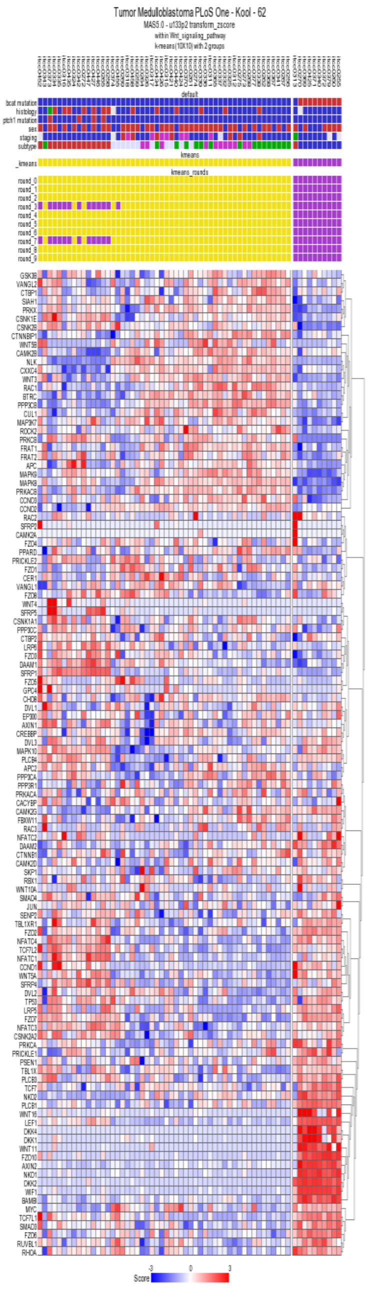


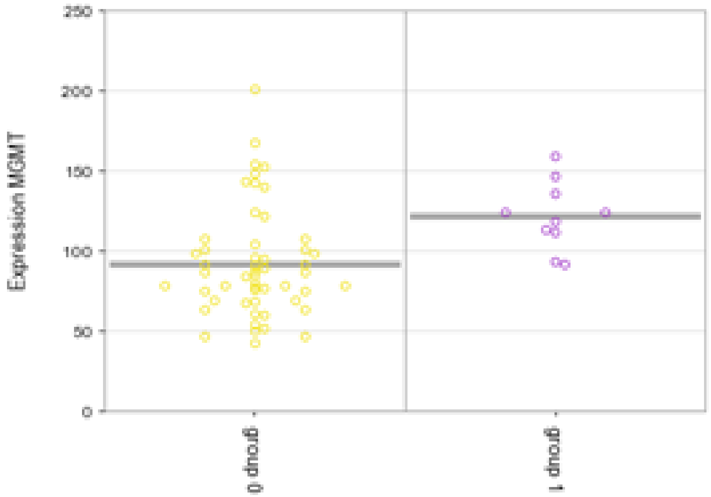


P=9.4e-3

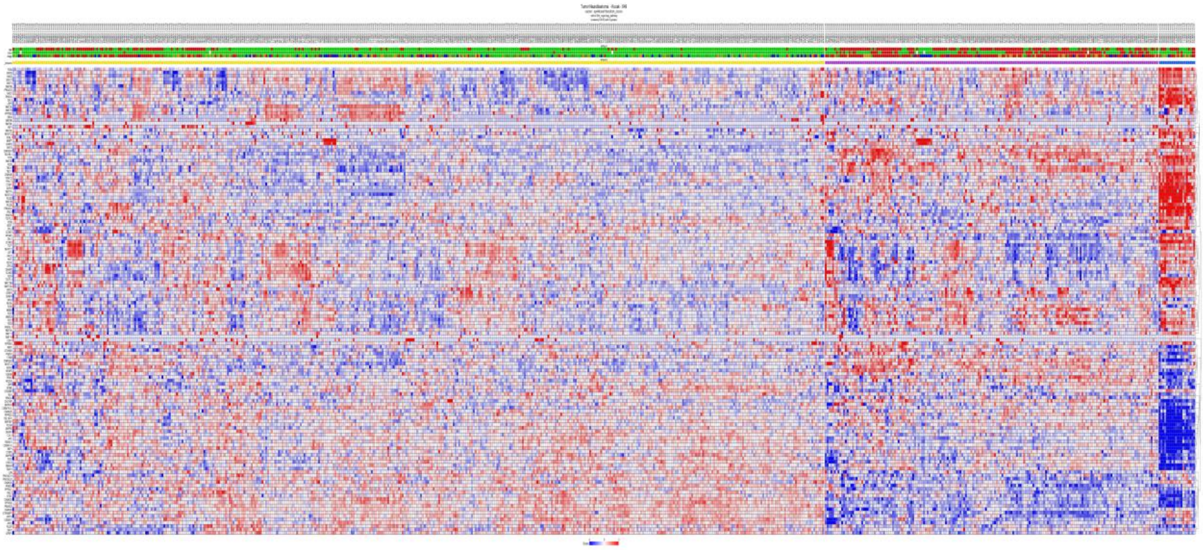

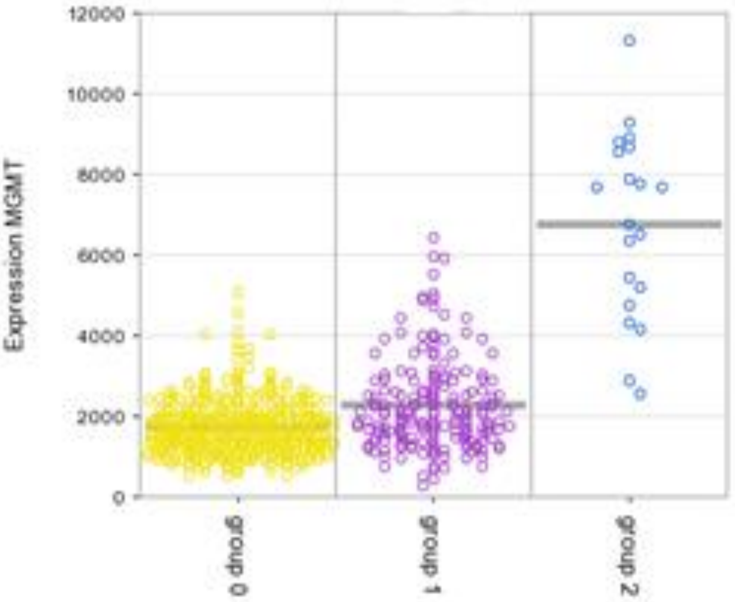
Expression MGMT
0
2000
4000
6000
8000
10000
12000
group 0
group 1
group 2

P=1.1e-95

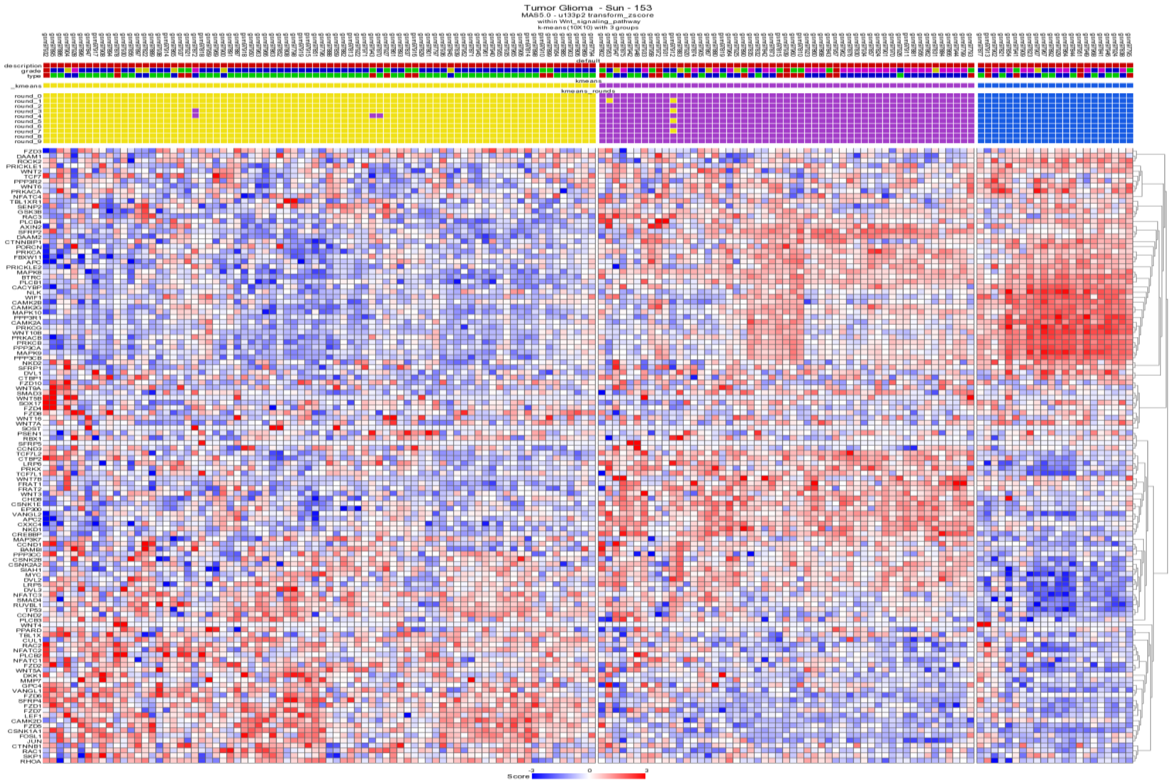
Tumor Glioma - Sun - 153
MAS5.0 - u133p2 transform_zscore
within Wnt_signaling_pathway
k-means(10X10) with 3 groups
Score

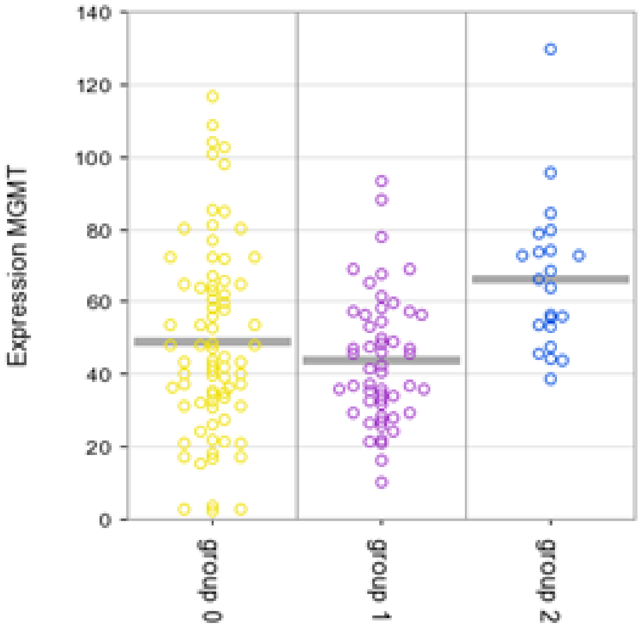
Expression MGMT
0
20
40
60
80
100
120
140
group 0
group 1
group 2

P=7.4e-4

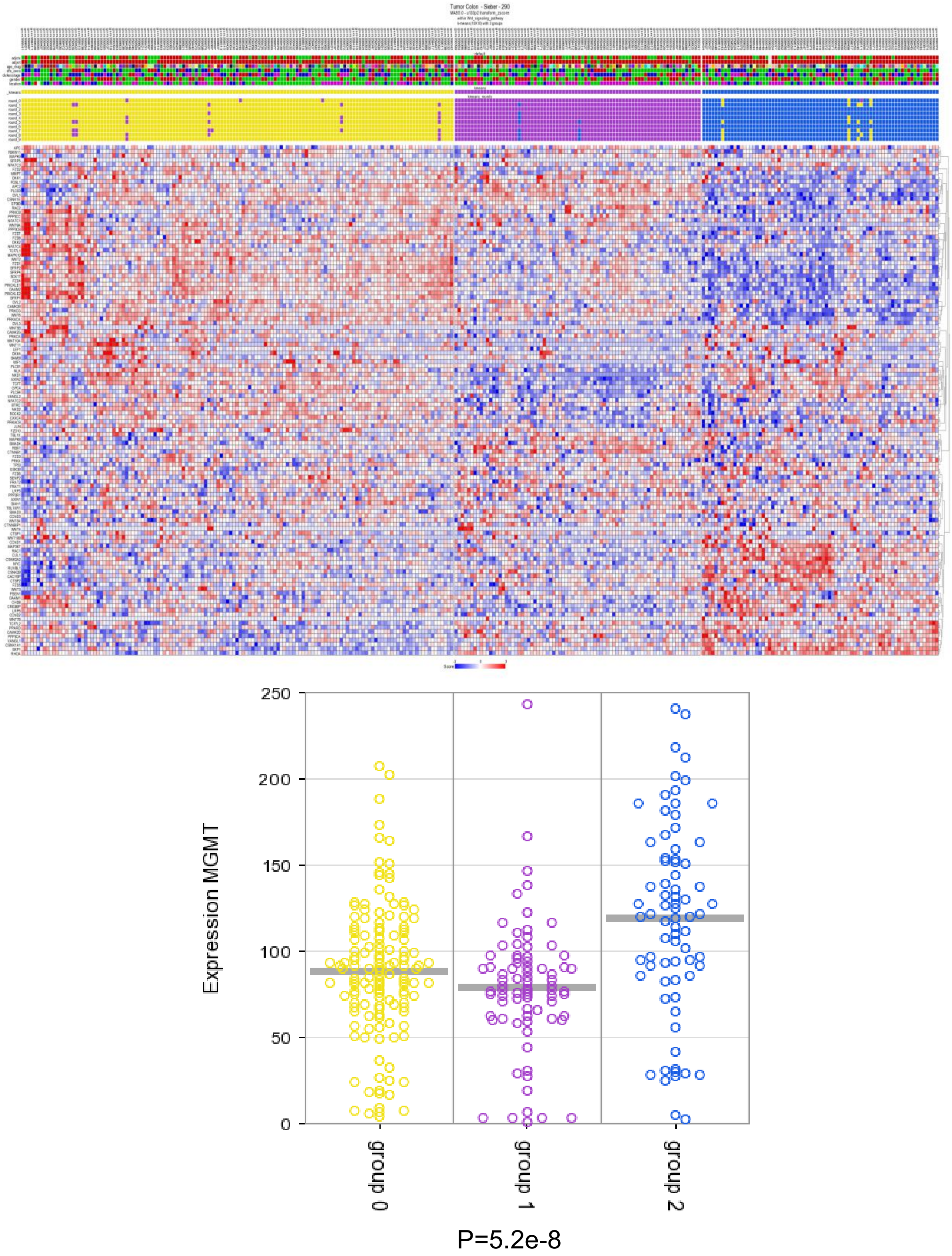


**Supplementary figure 2.** Unsupervised k-means clustering of genes associated with Wnt signalling correlated to MGMT expression. **(a)** medulloblastoma, **(b)** neuroblastoma, **(c)** glioma and **(d)** colon cancer.

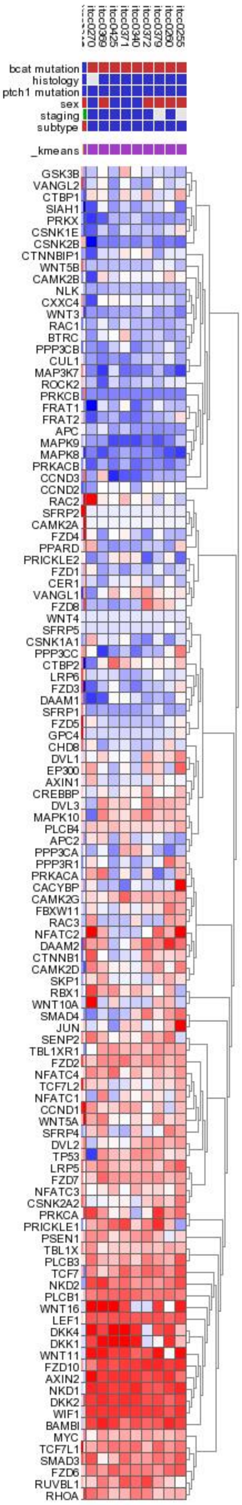
bcat mutation
histology
ptch1 mutation
sex
staging
subtype
_kmeans
GSK3B
VANGL2
CTBP1
SIAH1
PRKX
CSNK1E
CSNK2B
CTNNBIP1
WNT5B
CAMK2B
NLK
CXXC4
WNT3
RAC1
BTRC
PPP3CB
CUL1
MAP3K7
ROCK2
PRKCB
FRAT1
FRAT2
APC
MAPK9
MAPK8
PRKACB
CCND3
CCND2
RAC2
SFRP2
CAMK2A
FZD4
PPARD
PRICKLE2
FZD1
CER1
VANGL1
FZD8
WNT4
SFRP5
CSNK1A1
PPP3CC
CTBP2
LRP6
FZD3
DAAM1
SFRP1
FZD5
GPC4
CHD8
DVL1
EP300
AXIN1
CREBBP
DVL3
MAPK10
PLCB4
APC2
PPP3CA
PPP3R1
PRKACA
CACYBP
CAMK2G
FBXW11
RAC3
NFATC2
DAAM2
CTNNB1
CAMK2D
SKP1
RBX1
WNT10A
SMAD4
JUN
SENP2
TBL1XR1
FZD2
NFATC4
TCF7L2
NFATC1
CCND1
WNT5A
SFRP4
DVL2
TP53
LRP5
FZD7
NFATC3
CSNK2A2
PRKCA
PRICKLE1
PSEN1
TBL1X
PLCB3
TCF7
NKD2
PLCB1
WNT16
LEF1
DKK4
DKK1
WNT11
FZD10
AXIN2
NKD1
DKK2
WIF1
BAMBI
MYC
TCF7L1
SMAD3
FZD6
RUVBL1
RHOA

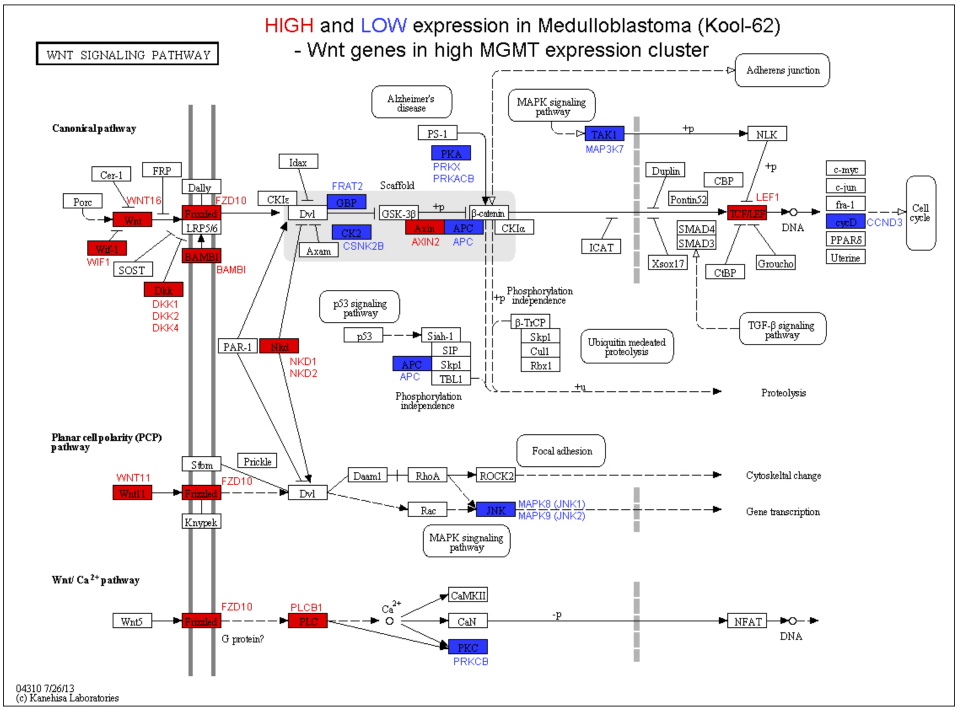
HIGH and LOW expression in Medulloblastoma (Kool-62)
- Wnt genes in high MGMT expression cluster
WNT SIGNALING PATHWAY
Canonical pathway
Planar cell polarity (PCP) pathway
Wnt/ Ca2+ pathway
WNT16
FZD10
WIF1
BAMBI
DKK1
DKK2
DKK4
NKD1
NKD2
FRAT2
PRKX
PRKACB
MAP3K7
CSNK2B
AXIN2
APC
LEF1
CCND3
WNT11
MAPK8 (JNK1)
MAPK9 (JNK2)
PLCB1
PRKCB
Adherens junction
Alzheimer's disease
MAPK signaling pathway
p53 signaling pathway
Ubiquitin mediated proteolysis
TGF-β signaling pathway
Focal adhesion
Phosphorylation independence
Proteolysis
Cytoskeletal change
Gene transcription
Cell cycle
DNA
G protein?
04310 7/26/13
(c) Kanehisa Laboratories

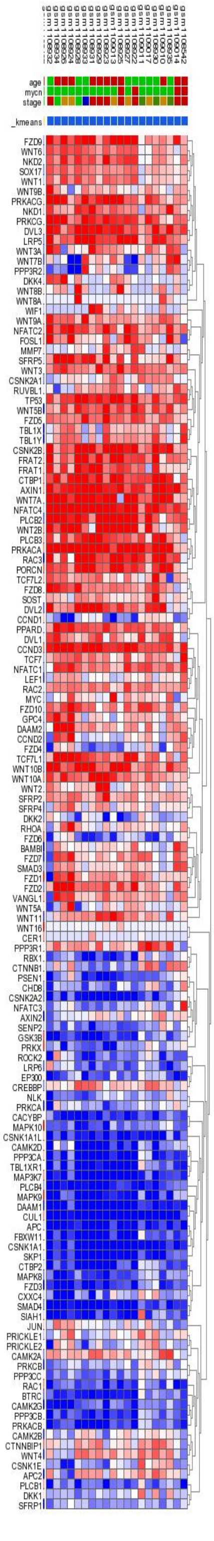
age
mycn
stage
_kmeans

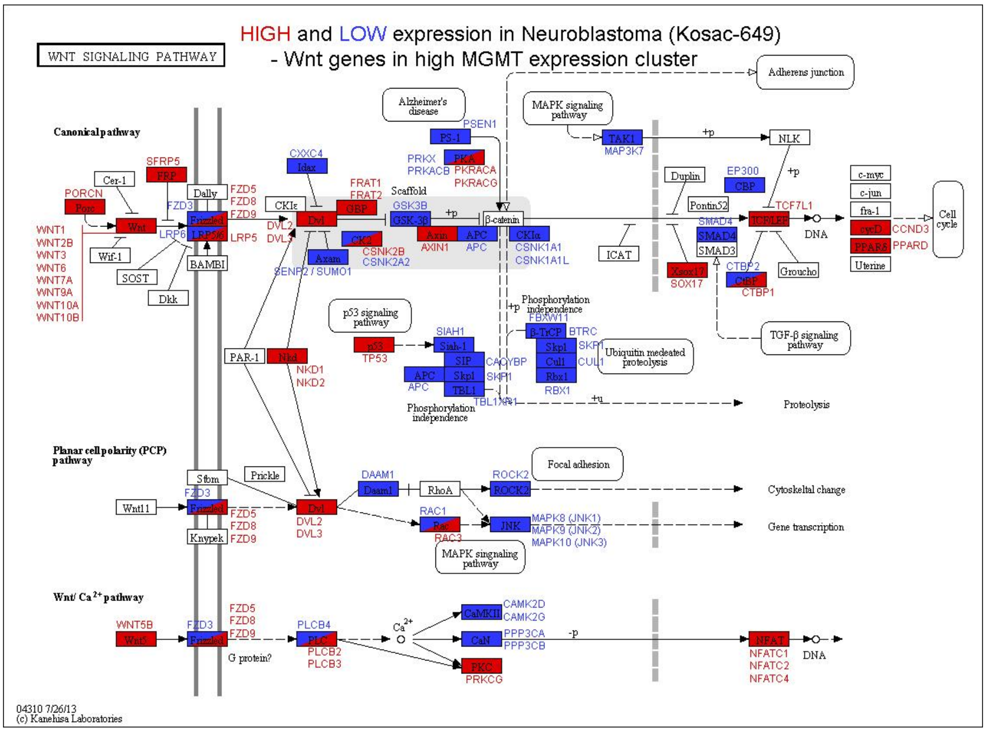
HIGH and LOW expression in Neuroblastoma (Kosac-649)
- Wnt genes in high MGMT expression cluster
WNT SIGNALING PATHWAY
Canonical pathway
Planar cell polarity (PCP) pathway
Wnt/ Ca2+ pathway
Adherens junction
Alzheimer's disease
MAPK signaling pathway
p53 signaling pathway
TGF-β signaling pathway
Ubiquitin mediated proteolysis
Focal adhesion
Cytoskeletal change
Gene transcription
Proteolysis
Cell cycle
DNA
04310 7/26/13
(c) Kanehisa Laboratories

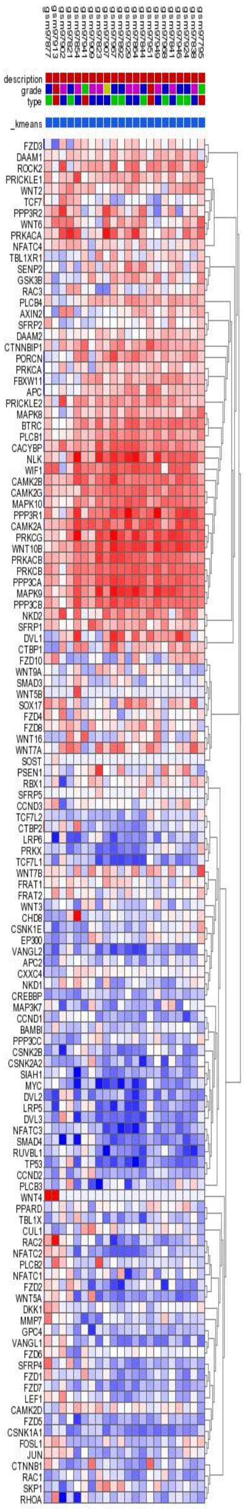

description
grade
type
_kmeans
FZD3
DAAM1
ROCK2
PRICKLE1
WNT2
TCF7
PPP3R2
WNT6
PRKACA
NFATC4
TBL1XR1
SENP2
GSK3B
RAC3
PLCB4
AXIN2
SFRP2
DAAM2
CTNNBIP1
PORCN
PRKCA
FBXW11
APC
PRICKLE2
MAPK8
BTRC
PLCB1
CACYBP
NLK
WIF1
CAMK2B
CAMK2G
MAPK10
PPP3R1
CAMK2A
PRKCG
WNT10B
PRKACB
PRKCB
PPP3CA
MAPK9
PPP3CB
NKD2
SFRP1
DVL1
CTBP1
FZD10
WNT9A
SMAD3
WNT5B
SOX17
FZD4
FZD8
WNT16
WNT7A
SOST
PSEN1
RBX1
SFRP5
CCND3
TCF7L2
CTBP2
LRP6
PRKX
TCF7L1
WNT7B
FRAT1
FRAT2
WNT3
CHD8
CSNK1E
EP300
VANGL2
APC2
CXXC4
NKD1
CREBBP
MAP3K7
CCND1
BAMBI
PPP3CC
CSNK2B
CSNK2A2
SIAH1
MYC
DVL2
LRP5
DVL3
NFATC3
SMAD4
RUVBL1
TP53
CCND2
PLCB3
WNT4
PPARD
TBL1X
CUL1
RAC2
NFATC2
PLCB2
NFATC1
FZD2
WNT5A
DKK1
MMP7
GPC4
VANGL1
FZD6
SFRP4
FZD1
FZD7
LEF1
CAMK2D
FZD5
CSNK1A1
FOSL1
JUN
CTNNB1
RAC1
SKP1
RHOA


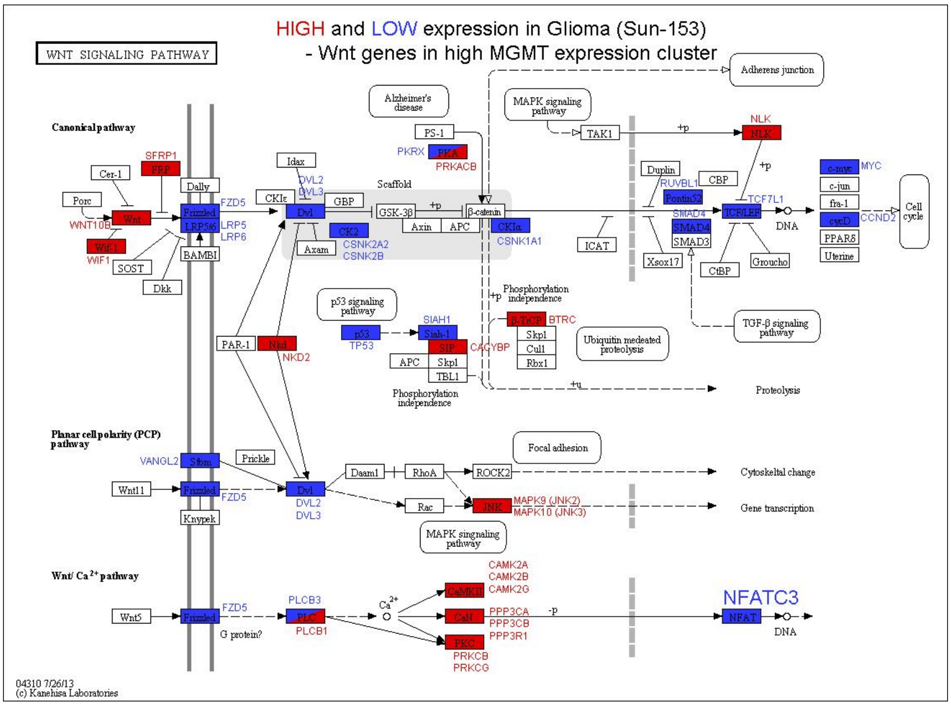

HIGH and LOW expression in Glioma (Sun-153)
- Wnt genes in high MGMT expression cluster
WNT SIGNALING PATHWAY
Canonical pathway
Planar cell polarity (PCP) pathway
Wnt/ Ca2+ pathway
Adherens junction
MAPK signaling pathway
Alzheimer's disease
p53 signaling pathway
Ubiquitin mediated proteolysis
TGF-β signaling pathway
Focal adhesion
Phosphorylation independence
Proteolysis
Cytoskeltal change
Gene transcription
Cell cycle
DNA
NFATC3
04310 7/26/13
(c) Kanehisa Laboratories

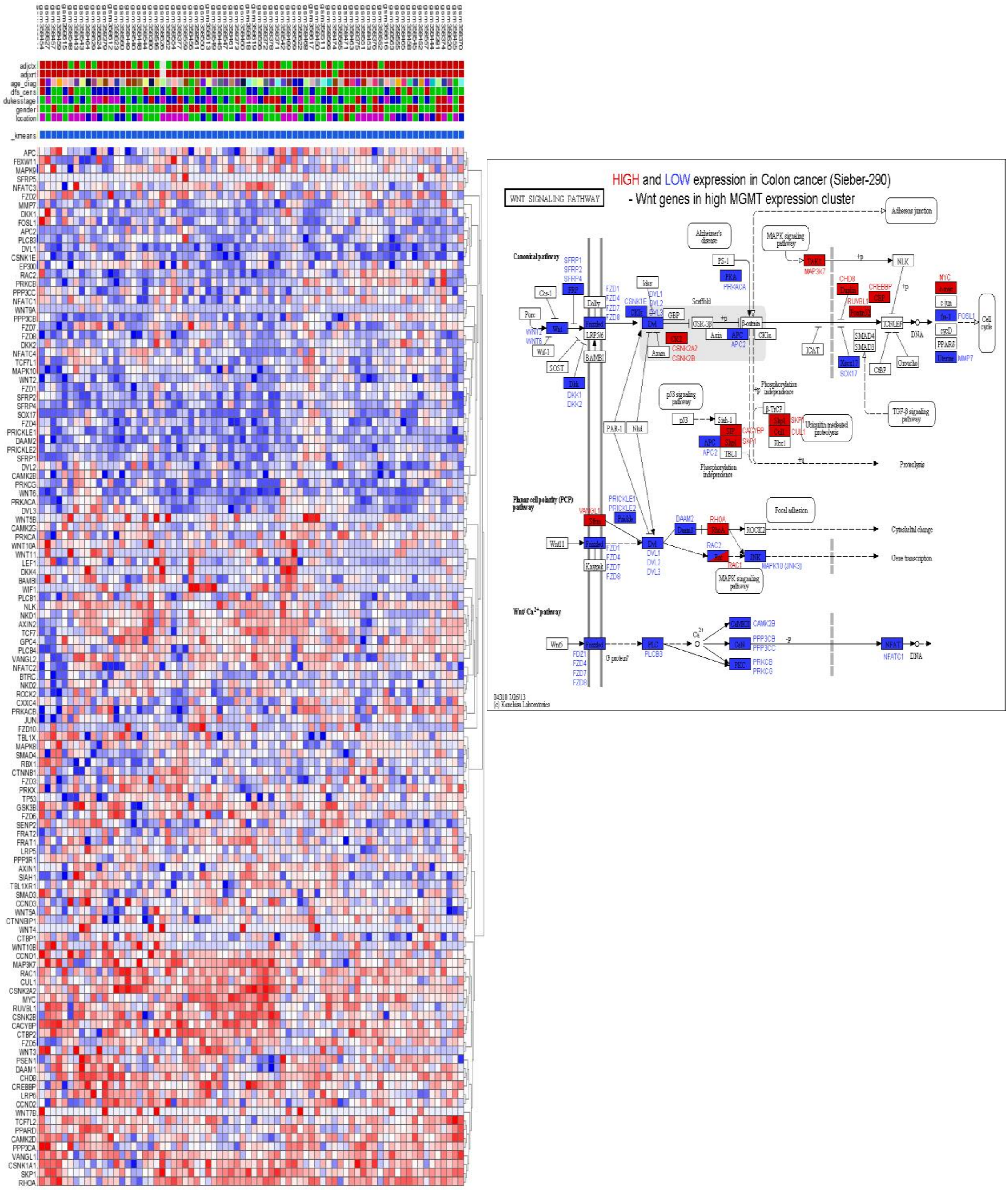


**Supplementary figure 3.** Heatmaps of relative gene expression of genes associate with Wnt signalling that correlate positive (red) or negative (blue) with high MGMT expression and their localization in the Wnt signalling pathway **(a)** medulloblastoma, **(b)** neuroblastoma, **(c)** glioma and **(d)** colon cancer.

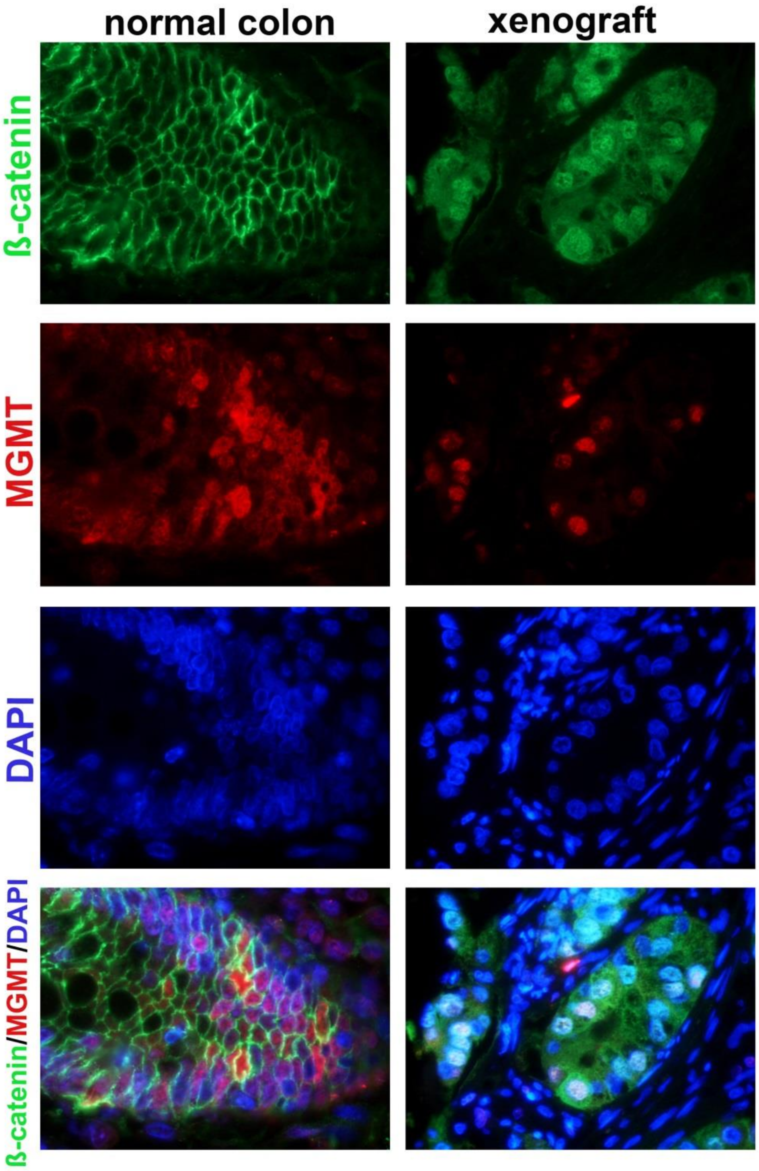


**Supplementary figure 4.** β-catenin and MGMT expression in normal colon and HT29 colon carcinoma xenograft. Shown are β-catenin (green), MGMT (red), DAPI (blue) and overlays (bottom).

[1]ACTCCAGCTCTTGGCTCCAACATTTACAGGAACACTGGGACTAAGAAAATCAATTGTGTTAATCAGCAAAACCCTCCACTTGA
GATGCTATCCTAAAAGCAAACTATATGTAAAACACTTAACGTGGGGAGCTCTGTAATATGTTTCTTTTGTTACACGACCATCAC
ACATTTGTCCGGAGTTTGGGGCTTTCAAACCACATAAGGTTTTATGTGATCGTTATAAAATCCCTTCATTTCCCAAAGGTAGGG
TAGTAAGGATTAAGTTTGACTACTGAAGGCAGCTTTGTTGTAAGTGGTCTCTAATGTCACAAAAGTAGCTTGGGAAGACAGGC
TTATTAAACAAATTTCTTGGGTTGCAAATCATTAGAAGCAATCGCATGTCATGTTCATTTGCTTAACCCGAAAGGTATCCATTT
GGATGTTTAAAGAAACTATGCAGATTAACATTGTTCCCAGTGCCCACAAGAAAGCTGGCTGATCTTAAAGGAAATCCAGGGAG
CTCCTTGAGGAAGTCGGACTGCAGTTGAGCATACCCTTTTGTGGTTTTCCCACCTCTTTTGTAAAAAGTTCTTCATAGGAATCTG
TGTCTGGAGGAAAAGGGGTTGTGATTTGTTAATCTGAAGATGGGAGACCTCAAAGGAATCTCACCCAACCAGTCCTGCACTGT
CATCCTATGAACAGAGAAGTCAGGTGGTTGCTCAGTGAGGGGCAGGGCTGGCACCACATCCGAGCCTCCCTGTCCTGTGTTCC
TTACTCTTCAGTGGACCCGGCGACCTCCCGGCTTTGGAGCCATCCTGTGTTCCTTTAACAGTAGGACGAACACACAGGCGATGA
TGAGATTGTAATGGACAAGGGGGAGAGGCGTGGGGAGCTCTTGATGAAAACTTGGGTTTTTGAAATTACCATGATGAAGGG
AGGAGCTTGCACCAGCAGGTGTAAGGAGTGTGAGTTTGAGTTCTGCTCATCAGACAGCCTAAGCAGTCACATCCTTGAATCCT
GGTCTTTTGCAGGCCAGTCCTTGGTCAGGTGGTGGCCTTTTTCCTCTTTCCCCTCTGCACTGAGACCCTTATTTGGCTCCTGATCC
TGGAGCCCCTGACACAGTCCTTACCACTGAAGTAGCTGTGGCCCTTCACATAGTTCTTCCCACCACGCCAGCAAGGGGACTGA
GTTCTTTCAGTACAGCCAGGGCCAGGTCCACACATCTCCCTGGGAAGCCAGCACTTTCTCTCGTGGACCGCCTCCCCATTCCCA
CAGGGCCCTCCTAAAGGGCCTTTGGATGACCACATTGGCCTTTCTTTCCTAGGGTGGGGAATTAGAGATGCTAAGACTGGCCA
GTCTCTTTTTCTTAAGAGTTTAGGGATTCGTCTTAAAATATTATTCAGATAAACGTATGAGTCATGTGTTAACTGCAAAACTACT
TAACAGCACACTTGTTAGAATATCAGTCGGCACTCTGATTCCATTTCGGCATTGTTTTTCCTTTGGTTCTATTAAGATCCCGGTT
CTAACTGGGTCCTGGTAGTAACAGGCTGTAAGTGGCCAGGTGTATAGCATTATTTCTTAGTAGTGTGGTGTGGTGCTGGTGGTT
TATTTTCCCTATGCACACATGCCCTAATATGGAAGGATAGTTTGTACACACGTAGGGTACGTTATCATGAGACTCCTTTACAGA
GTATGAAACTGAGGATTAGAGAAGTCAGACGAGTTTCCCGAAGTCTTATAGAAGGCTAATGGAAAAAACCAGAGCCAAGGGC
AAACATGTTTCATCTGTCCTTCCACATTAGTTTAAATGTGAATGGCTGCTTGGAACACAGTGTTGAGAAGGATTCCGAGGCTGT
GTCCAGGTTCAGTGGGGAAAAAGTGCCTTGATTTGACCATTATATCGTGTACTCAGGGCCTAGTTTATTTCCCACAAGCAGCTA
TTTAAAACATGTCTCATCCAAGGCAGGGTCGGGGTGGGGAACCTCAGAGGATCAATGATGCAGCCTGTATTGTCACCAGGGCT
CATCCCAAAGCAAATCCTGCTGAAGTTCTCCTCTGAACAACCCTTATCACTCATGCTAATATGTTGGATCCTGCAAGTCACAAA
TACGAAGGTATGAGCATTGGCCGAATCCCAACAGGGAAACTTCACCGGGAAGGTGAAGACCCCTTTCCATTAAGATAACAAAC
AGAACCTAGAAGGCCCGTAGCATGGTGGCTTAGAACATGTGGGCCTTGGATGGGATCCTGCCAAGGGGTGTGTGACCTCTCCG
AAGCCTCCAGGGATGATACTACTCCCTGGGGTGCTTATGCCAACCACGTTAGAGACAATGGTTTCTGTACCCATTGCCTGGGGC
TGCCATAATAAAGTGCCACACACTGAGTGGCTTAAAACAACAGAAACCTATTGTCTCACACTTCCGGGGGCCAGAAGTTTGAA
ACCCAGGTGTGTTAGGATCCTGCTCCCTCTGAAGGCTCCAGGGAAGAGTGTCCTCTGCTCCCTCCGAAGGCTCCAGGGAAGGG
TCTGTCCTCTTAGGCTTCTGGTGGCTTGCAGGTGCAGCCCTCCAATCCTCCTCCCCAAGCGGCCTTCTGCCTATAAGGACACGA
GTCATACTGGATGAGGGGCCCACTAATTGATGGCTTCTGTAAAGTCCCCATCTCCAAATAAGGTCACATTGTGAGGTACTGGG
AGTTAGGACTCCAACATAGCTTCTCTGGTGGACACAATTCAACTCCTAATAACGTCCACACAACCCCAAGCAGGGCCTGGCAC
CCTGTGTGCTCTCTGGAGAGCGGCTGAGTCAGGCTCTGGCAGTGTCTAGGCCATCGGTGACTGCAGCCCCTGGACGGCATCGC
CCACCACAGGCCCTGGAGGCTGCCCCCACGGCCCCCTGACAGGGTCTCTGCTGGTCTGGGGGTCCCTGACTAGGGGAGCGGCA
CCAGGAGGGGAGAGACTCGCGCTCCGGGCTCAGCGTAGCCGCCCCGAGCAGGACCGGGATTCTCACTAAGCGGGCGCCGTCC
TACGACCCCCGCGCGCTTTCAGGACCACTCGGGCACGTGGCAGGTCGCTTGCACGCCCGCGGACTATCCCTGTGACAGGAAAA
GGTACGGGCCATTTGGCAAACTAAGGCACAGAGCCTCAGGCGGAAGCTGGGAAGGCGCCGCCCGGCTTGTACCGGCCGAAGG
GCCATCCGGGTCAGGCGCACAGGGCAGCGGCGCTGCCGGAGGACCAGGGCCGGCGTGCCGGCGTCCAGCGAGGATGCGCAGA
CTGCCTCAGGCCCGGCGCCGCCGCACAGGGCATGCGCCGACCCGGTCGGGCGGAACACCCCGCCCCTCCCGGGCTCCGCCCC

AGCTCCGCCCCCGCGCGCCCCGGCCCCGCCCCCGCGCGCTCTCTTGCTTTTCTCAGGTCCTCGGCTCCGCCCCGCTCTAGACCCC
GCCCCACGCCGCCATCCCCGTGCCCCTCGGCCCCGCCCCCGCGCCCCGGATATGCTGGGACAGCCCGCGCCCCTAGAACGCTTT
GCGTCCCGACGCCCGCAGGTCCTCGCGGTGCGCACCGTTTGCGACTTGGTGAGTGTCTGGGTCGCCTCGCTCCCGGAAGAGTGC
GGAGCTCTCCCT$^{3600}$

TCF-4E/LEF-1

TCF-4

**Supplementary figure 5.** 5´-upstream region of the human MGMT gene (-3500/+1) showing the location of putative Tcf/Lef binding sites.

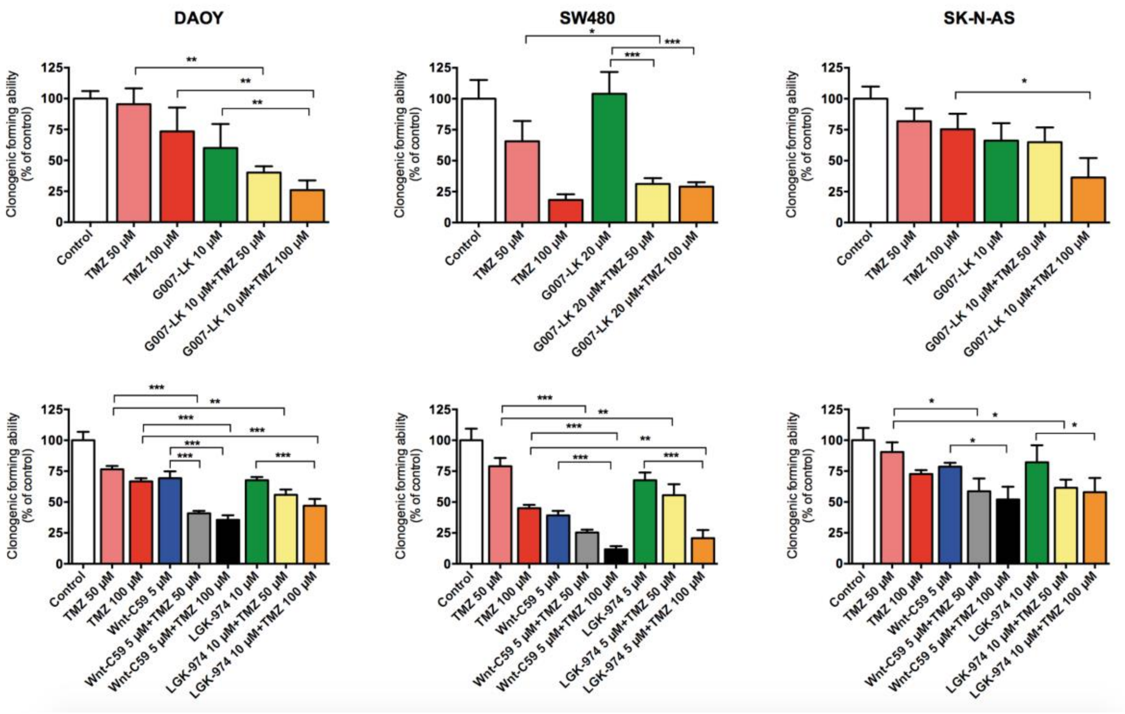


**Supplementary figure 6.** Clonogenic capacity of DAOY, SW480 and SK-N-AS cells treated with the indicated concentrations of Wnt-C59, LGK-974 or G007-LK and temozolomide. Cells were then incubated in drug-free medium for 7-14 days and colonies (>75 cells) with 50% plate efficiency (PE) were counted. Each experimental point was performed in triplicate. The experiment was repeated with similar results. Values are mean ± s.d. One-way ANOVA for G007-LK in DAOY and SW480: P<0.0001, SK-N-AS P=0.0012. One-way ANOVA for LGK974 and Wnt-C59 in DAOY, SW480 and SK-N-AS: P<0.0001. Bonferroni post test: *P<0.05, **P<0.01, ***P<0.001).

**a.**

**Active β-catenin (92 kDa)**

Medulloblastoma

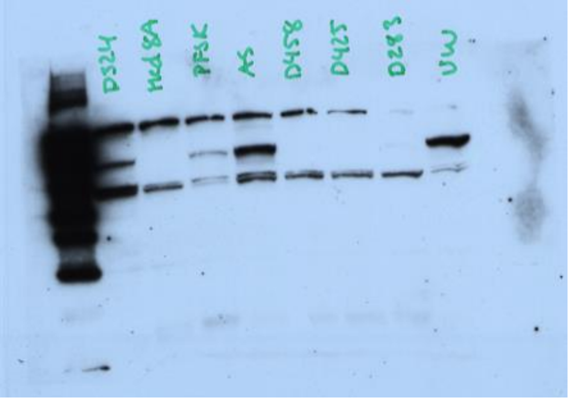


Colon

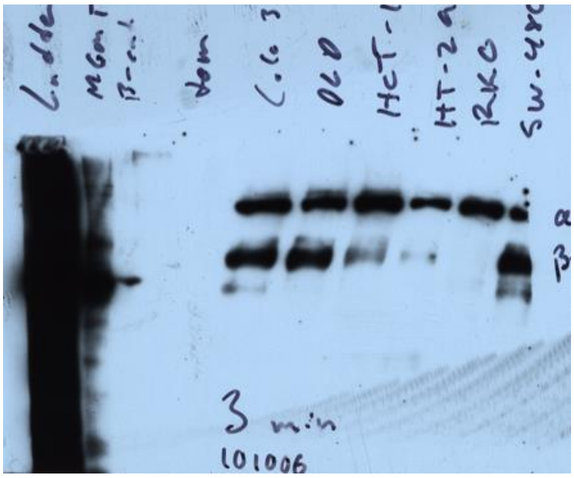


Glioma

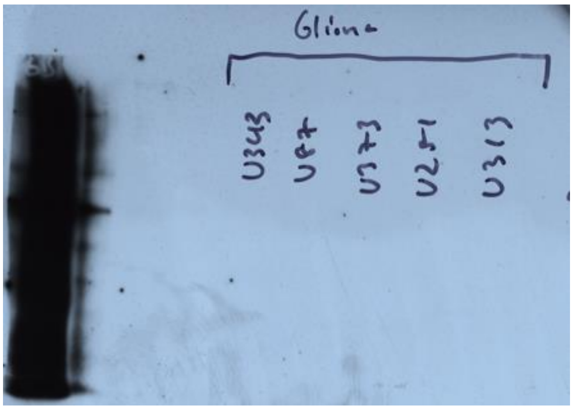

Glioma (T98G)

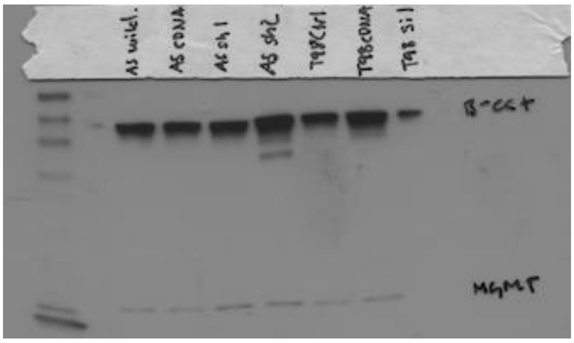


Neuroblastoma

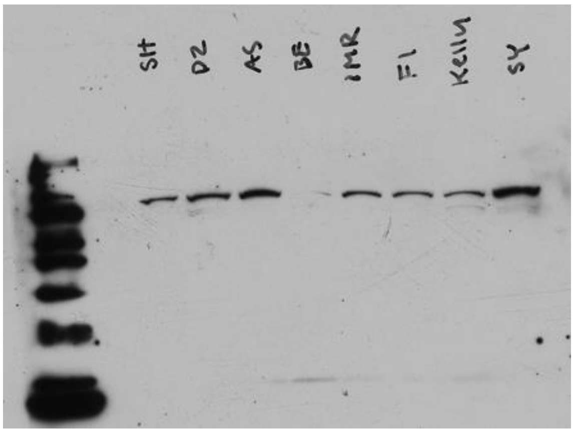


**β-actin (45 kDa)**

Medulloblastoma

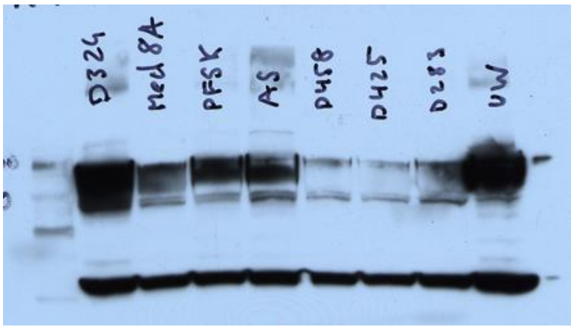

Colon

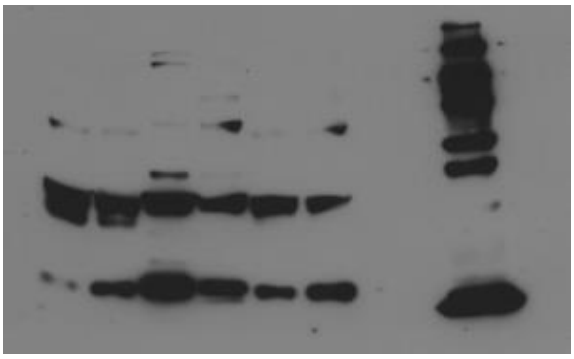

Glioma

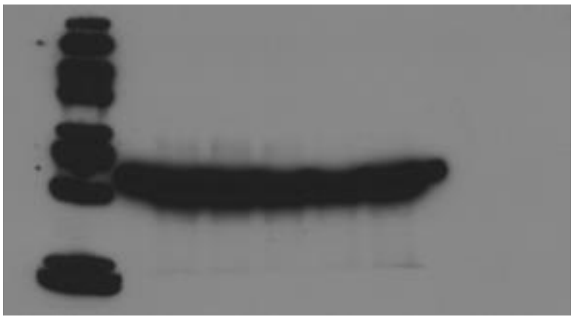

Glioma (T98G)

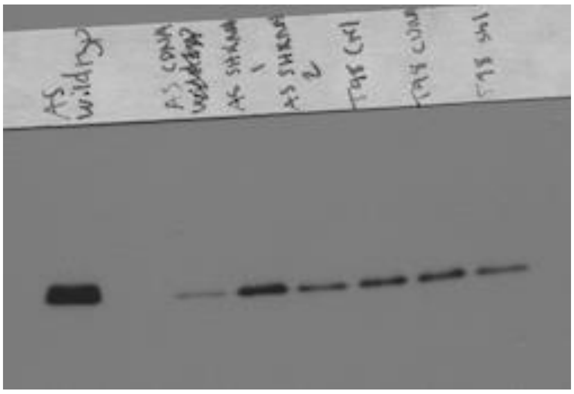

Neuroblastoma

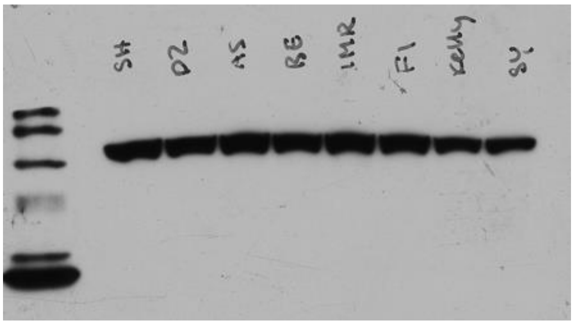

**Axin2 (95 kDa)**

Medulloblastoma

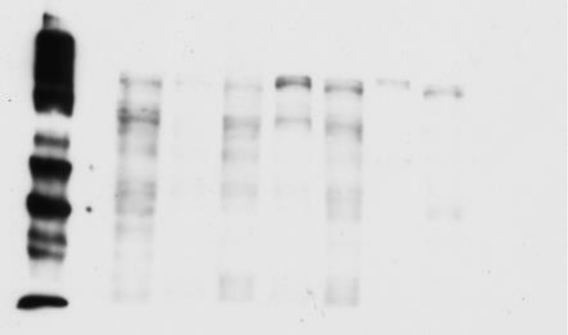

Colon

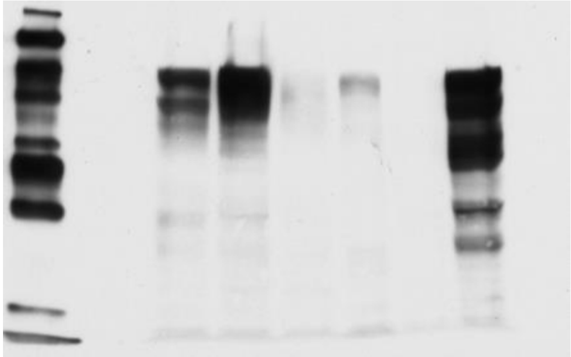

Glioma

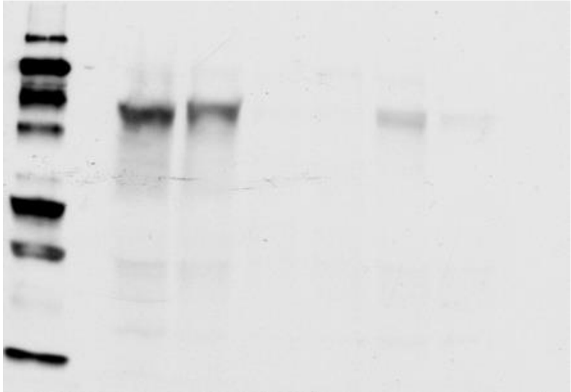

Neuroblastoma

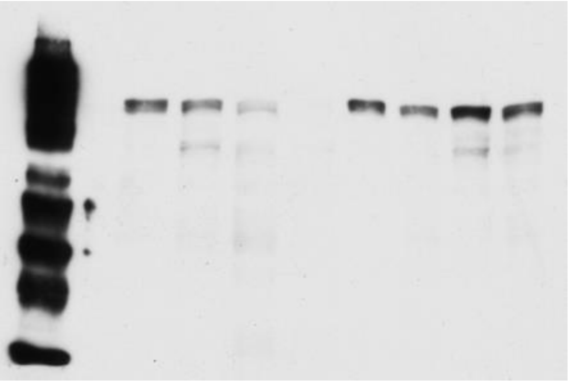

**MGMT (21 kDa)**

Medulloblastoma

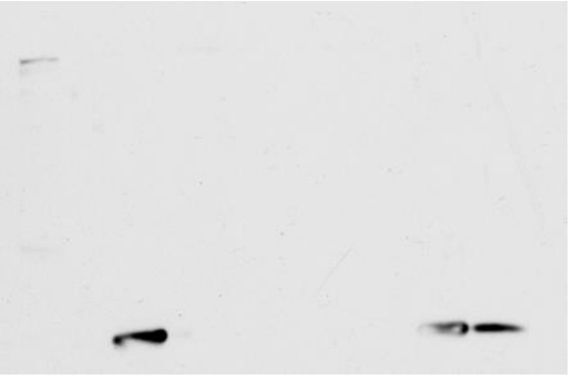

Colon

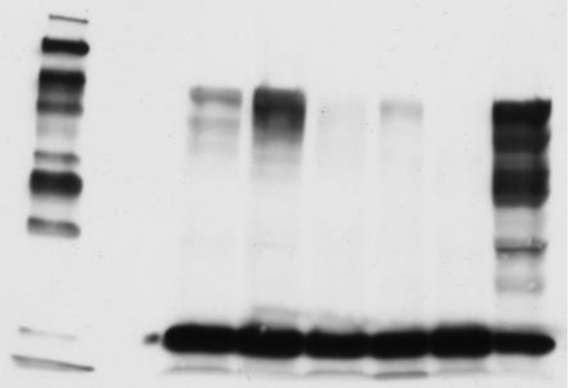

Glioma

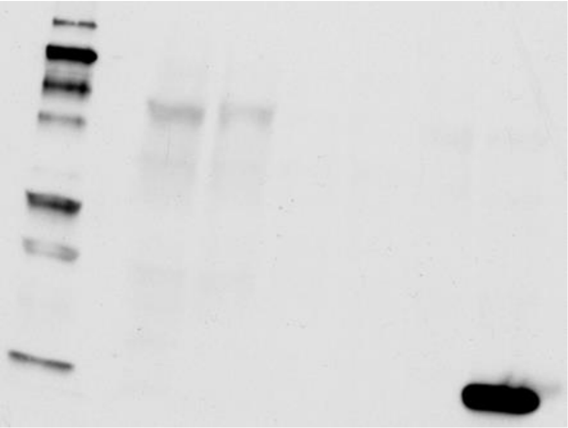

Neuroblastoma

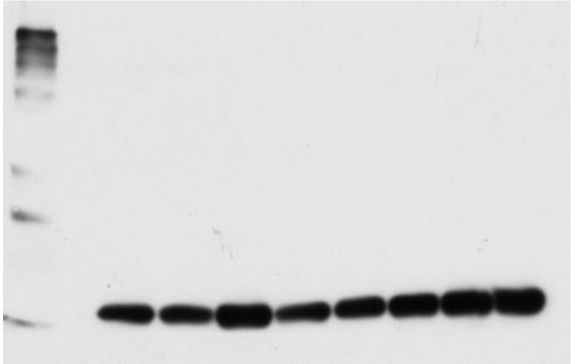

**GAPDH (37 kDa)**

Medulloblastoma

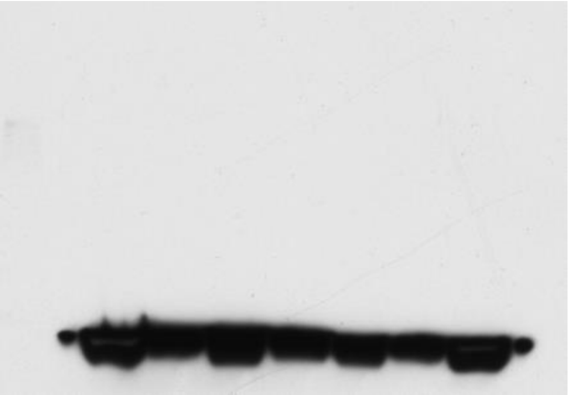

Colon

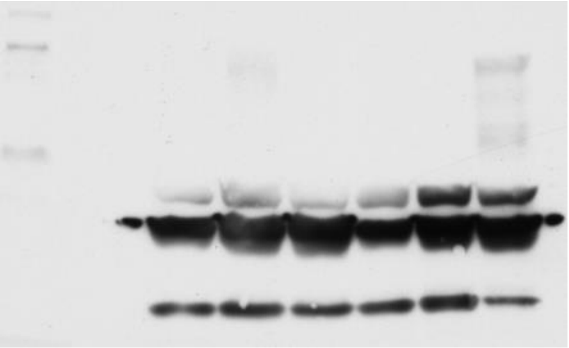

Glioma

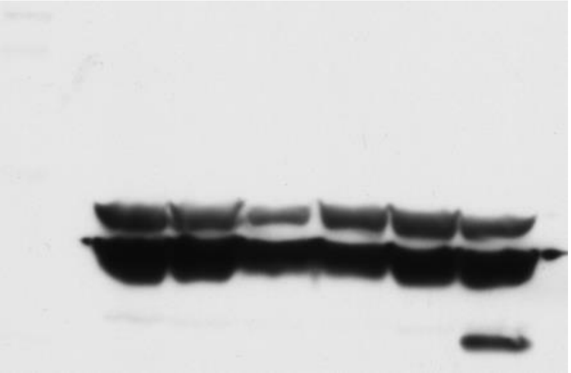

Neuroblastoma

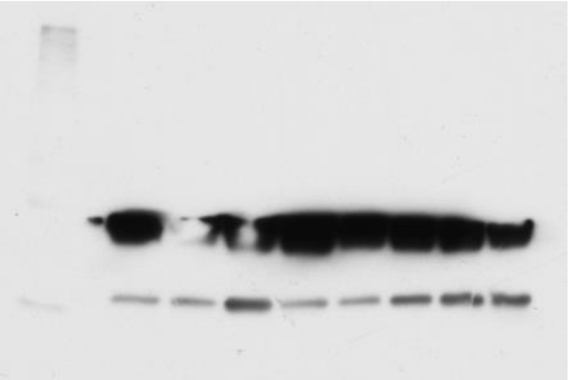

**b.**

**Active β-catenin (92 kDa)**

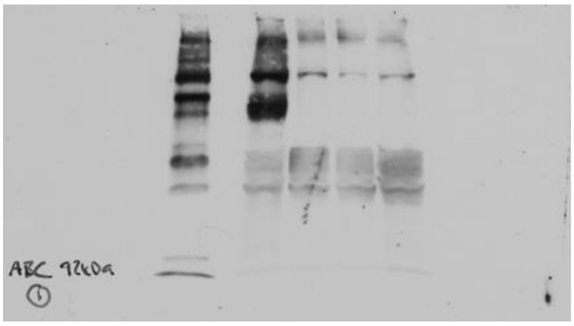


**Axin2 (95 kDa)**

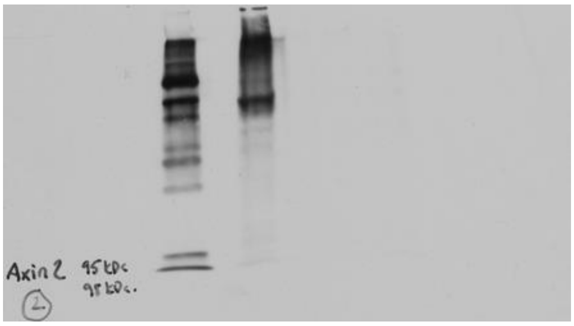


**MGMT (21 kDa)**

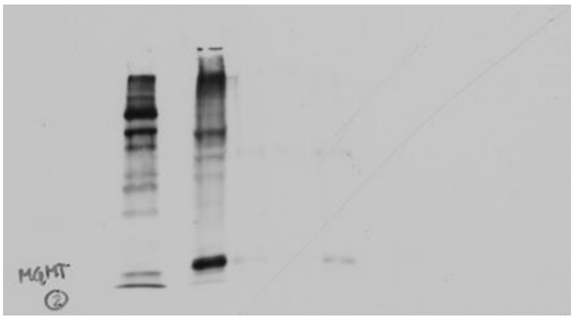


**GAPDH (37 kDa)**

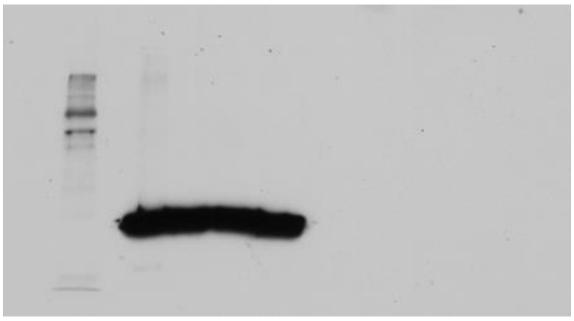

**c.**

**Active β-catenin (92 kDa) and MGMT (21 kDa)**

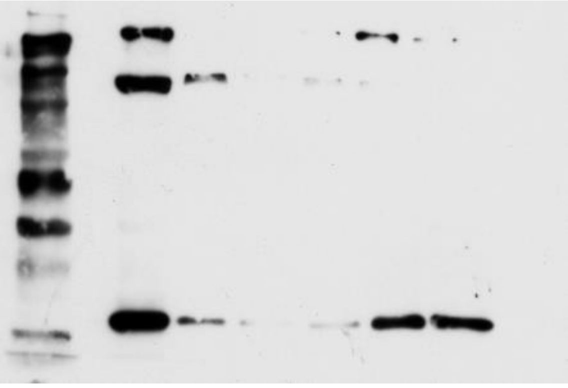

**GAPDH (37 kDa)**

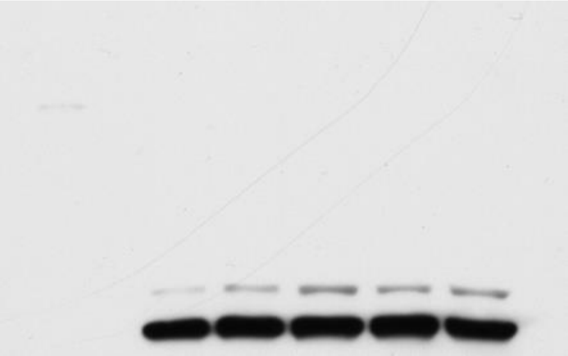

**d.**

**MGMT (21 kDa) and β-actin (45 kDa)**

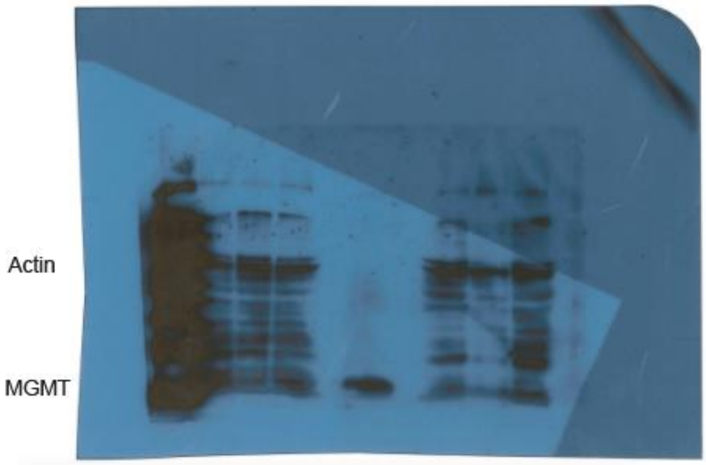

**e.**

**Active β-catenin (92 kDa)**

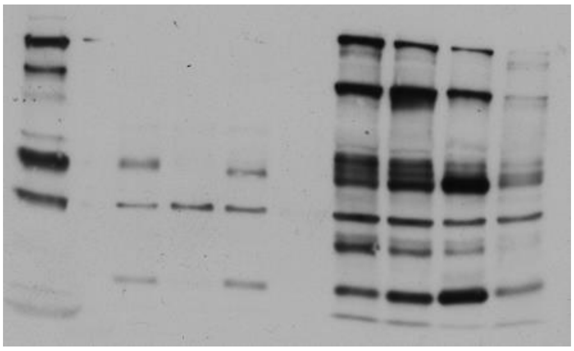

**MGMT (21 kDa)**

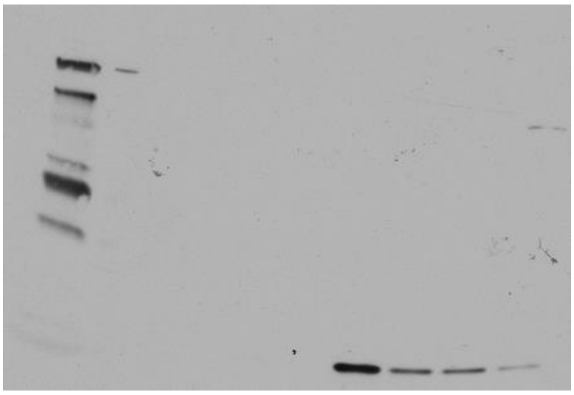

**GADPH (37 kDa)**

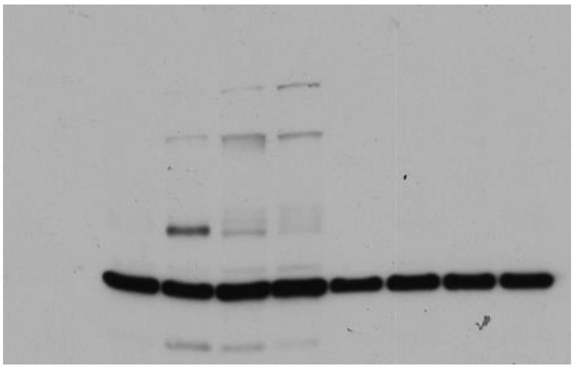

**Supplementary figure 7**. Full western blots **(a)** Fig. 1d, **(b)** Fig. 2b, (**c**) Fig. 3c, (**d**) Fig. 5b and (**e**) Fig. 5d.

**Effect of celecoxib in combination with chemotherapeutics in medulloblastoma/supratentorial primitive neuroectodermal tumor cells in vitro.**

Combination index (CI) at $IC_{70}$, analyzed using the median-effect method of Chou and Talalay. Synergism and antagonism are defined as a CI mean statistically significantly lower/higher than 1 with one-sample t-test ($P<0.05$). The studies were designed with a fixed molar ratio between the drugs, intended to be equipotent.

| | DAOY | | D283 MED | | PFSK-1 | |
|---|---|---|---|---|---|---|
| | **CI ± s.d.** | **Effect** | **CI ± s.d.** | **Effect** | **CI ± s.d.** | **Effect** |
| CCI-779 | 0.51 ± 0.18 | Synergistic | 0.45 ± 0.15 | Synergistic | 0.86 ± 0.10 | Additive |
| Cisplatin | 2.45 ± 0.54 | Antagonistic | 1.40 ± 0.11 | Antagonistic | 1.24 ± 0.091 | Antagonistic |
| Cyclophosphamide | 0.93 ± 0.19 | Additive | 0.75 ± 0.17 | Additive | 0.70 ± 0.18 | Additive |
| Doxorubicin | 0.61 ± 0.065 | Synergistic | 0.58 ± 0.21 | Synergistic | 0.42 ± 0.088 | Synergistic |
| Irinotecan | 0.94 ± 0.15 | Additive | 0.96 ± 0.11 | Additive | 0.93 ± 0.10 | Additive |
| Rapamycin | 0.81 ± 0.081 | Synergistic | 0.76 ± 0.21 | Additive | 1.00 ± 0.29 | Additive |
| Temozolomide | 0.75 ± 0.14 | Synergistic | 0.80 ± 0.075 | Synergistic | 0.93 ± 0.10 | Additive |
| Vincristine | 1.40 ± 0.59 | Additive | 0.55 ± 0.15 | Synergistic | 0.94 ± 0.19 | Additive |

**Supplementary Table 1.** Effect of celecoxib in combination with chemotherapeutic drugs in medulloblastoma/supratentorial primitive neuroectodermal tumor cells *in vitro*.
